# AllesTM: predicting multiple structural features of transmembrane proteins

**DOI:** 10.1186/s12859-020-03581-8

**Published:** 2020-06-12

**Authors:** Peter Hönigschmid, Stephan Breimann, Martina Weigl, Dmitrij Frishman

**Affiliations:** Department of Bioinformatics, Wissenschaftszentrum Weihenstephan, Technische Universität München, Maximus-von-Imhof-Forum 3, 85354 Freising, Germany

**Keywords:** Protein evolution, Protein structure prediction, Transmembrane proteins, Machine learning

## Abstract

**Background:**

This study is motivated by the following three considerations: a) the physico-chemical properties of transmembrane (TM) proteins are distinctly different from those of globular proteins, necessitating the development of specialized structure prediction techniques, b) for many structural features no specialized predictors for TM proteins are available at all, and c) deep learning algorithms allow to automate the feature engineering process and thus facilitate the development of multi-target methods for predicting several protein properties at once.

**Results:**

We present AllesTM, an integrated tool to predict almost all structural features of transmembrane proteins that can be extracted from atomic coordinate data. It blends several machine learning algorithms: random forests and gradient boosting machines, convolutional neural networks in their original form as well as those enhanced by dilated convolutions and residual connections, and, finally, long short-term memory architectures. AllesTM outperforms other available methods in predicting residue depth in the membrane, flexibility, topology, relative solvent accessibility in its bound state, while in torsion angles, secondary structure and monomer relative solvent accessibility prediction it lags only slightly behind the currently leading technique SPOT-1D. High accuracy on a multitude of prediction targets and easy installation make AllesTM a one-stop shop for many typical problems in the structural bioinformatics of transmembrane proteins.

**Conclusions:**

In addition to presenting a highly accurate prediction method and eliminating the need to install and maintain many different software tools, we also provide a comprehensive overview of the impact of different machine learning algorithms and parameter choices on the prediction performance.

AllesTM is freely available at https://github.com/phngs/allestm.

## Introduction

Starting with the seminal work of Qian and Sejnowski [1], only the sky seems to be the limit for the application of machine learning methods to sequence-based protein structure prediction. Over the years, as the variety of employed algorithms expanded (neural networks, support vector machines, random forests), so did the scope of prediction targets – from secondary structure, transmembrane regions, and signal peptides to solvent accessibility, crystallizability, contact maps, and interaction sites. The art of predictor design is not only in the construction of an optimal machine learning framework and rigorous training and testing procedures, but also in the meticulous choice of the relevant feature space. In each particular case, the latter step requires in-depth domain knowledge and almost unavoidably involves arbitrary decisions. Recent major advances in protein structure prediction accuracy, made apparent by the latest rounds of the CASP contest [2], can be attributed to the wide adoption of deep learning methods [3, 4], which are able to learn hierarchical features and infer input/output mappings directly from complex and noisy data (Goodfellow et al., 2016). One specific advantage of this group of methods is that they allow automating the feature engineering process and thus eliminate, or at least significantly alleviate, the arguably most time-consuming step in the development of bioinformatics prediction algorithms. This, in its turn, opens up the possibility of developing multi-target prediction methods, i.e. methods that predict a whole array of protein properties directly from input sequences or evolutionary profiles.

In this work, we explore this idea focusing on integral transmembrane proteins. In spite of its functional importance and biomedical relevance, this class of proteins has been, to some extent, neglected in the field of structural bioinformatics, mostly due to the sparsity of experimental data available for training algorithms. For example, the residue contact prediction field was initiated in 1994 [5], but it was not until 2007 that specialized contact prediction methods for membrane proteins were proposed [6, 7]. Likewise, the first attempts to predict protein solubility were made almost 30 years ago [8], while for membrane proteins the first predictor of solubility and other experimental properties was developed in 2008 [9]. For some structural features, such as flexibility, secondary structure, torsion angles or solvent accessibility, membrane protein-specific predictors are not available at all. Experience shows that, in many cases, applying methods originally trained on soluble proteins to predict the structural features of transmembrane proteins leads to inferior performance.

Here we present AllesTM, an integrated tool to predict several essential structural features of transmembrane proteins: residue depth in the membrane, flexibility, topology, three-state secondary structure, relative solvent accessibility (in the protein’s monomer form as well as bound in a complex), and the torsion angles φ and ψ. By combining state of the art machine learning algorithms, a modern dataset of known transmembrane protein structures, widely accepted evaluation methods and metrics as well as heavy automation of training and parameter selection we were able to either outperform or at least achieve a comparable performance to the currently leading methods in terms prediction accuracy. An additional practical benefit of AllesTM is that it eliminates the need to install and maintain many different software tools and allows for easy installation by minimizing dependencies and proper packaging.

## Results

### Z-coordinates

**Fig. 1:**
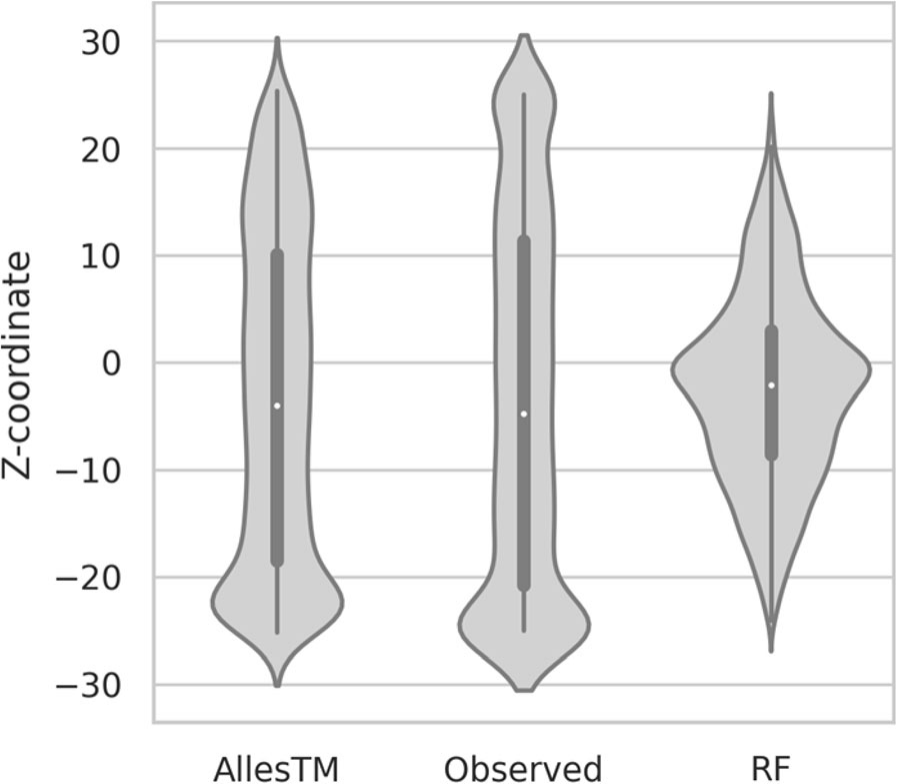
Distribution of the observed and predicted z-coordinates on the independent test dataset. Only the most basic (RF) and the most advanced method (AllesTM) are shown

The depth of a residue in the membrane is predicted as a continuous number, its distance from the membrane center, ranging from − 25 to + 25 Å (Fig. 1, S1 and S2 Figs). Significant deviations from the equal distribution of residue occurrence beyond the central portion of the transmembrane segment (i.e. outside of the range − 15 to + 15) can be explained as follows. The slightly higher occurrence of residues in the vicinity of the membrane boundaries (around − 15 and + 15) is apparently caused by interfacial helices oriented parallel to the membrane as well as reentrant regions. Minor ‘dents’ in the distribution at approximately − 20 Å and especially at 20 Å are due to the fact that in our data many amino acid chains begin (or end) at this distance from the membrane, and the protein N- or C-termini are more frequently positioned at 20 Å than at − 20 Å. Finally, peaks at − 25 Å and 25 Å are a trivial consequence of the fact that any values beyond this range are assigned these values.

While the z-coordinates are the only target where no other method is publicly available for comparison, several conclusions regarding the employed machine learning algorithm can be drawn from the performance overview shown in S1 Table. First, the accuracy increases with the receptive field of the method. The RF and the GBM with their limited receptive field achieve an MAE of 10.64 and 9.63, respectively, on the cross-validation dataset. The low performance of the RF is also visualized in Fig. 1, Fig. S1 and Fig. S2, showing that the predicted values deviate strongly from the expected distribution and are accumulated around a z-coordinate of 0. The conv method, which has a much wider receptive field due to its multiple layers, already achieves an MAE of 6.94 on the same data, while the dconv and LSTM approaches outperform the other models with MAEs of 6.02 and 4.99, respectively. The importance of a large receptive field indicates the depth of the residue on the membrane is influenced by long-range correlations between amino acid residues. At least for the cross-validation dataset, the MAE of the final method AllesTM (5.08) is slightly higher than the MAE of the LSTM model, but with a reduced MSE/RMSE. AllesTM produces results that resemble the observed distribution of z-coordinates quite well, especially when compared to the RF method (Fig. 1). All approaches without exception perform better on the independent test dataset than on the cross-validation dataset. This can have one of two reasons: either the independent test dataset is easier to predict, or the averaging of the five models derived from cross-validation led to an increased performance as the averaging procedure is equivalent to additional ensembling. The expected MAE from the final method AllesTM is 3.72 with an RMSE of only 6.32, which means that there are only a few large errors.

### Topology

AllesTM predicts not only the position of a residue with respect to the membrane as an absolute value, but also in which of the four types of segments the residue is situated in: inner and outer side of the membrane (In/Out), transmembrane segment (TMS), or reentrant region (RER). Table 1, S2 Table, Fig. S3 and Fig. S4 show the performance of the trained algorithms compared with MEMSAT-SVM, PolyPhobius and SCAMPI on the cross-validation as well as the independent test dataset. Similar to the z-coordinate prediction, a larger receptive field has a positive impact on the prediction performance, although the effect is generally less pronounced compared to the z-coordinate prediction and more prominent for the In and Out segments than for the TMS and RER segments. For example, the RF model reaches an AUC of 0.85 compared to an AUC of 0.9 for the LSTM model in the In segments, while for the TMS segments the AUC differs only by 0.01 (0.88 for the RF model and 0.89 for the LSTM model). Furthermore, in terms of accuracy AllesTM outperforms MEMSAT-SVM as well as PolyPhobius and SCAMPI. This holds true when not including RER segments into the benchmark, a class neither PolyPhobius nor SCAMPI can predict. Focusing on the classes In, Out, TMS, and RER separately, only for RER segments MEMSAT-SVM achieves a higher AUC value.

**Table 1:** Performance summary of all methods and predictions targets evaluated on the independent test dataset. Bold face indicates the best performing method. More detailed metrics, broken down to individual classes, can be found in the supplementary materials

	**Z-coordinates**	**Topology**	**Continuous flexibility**	**Two-state flexibility**		
	**MAE**	**ACC**	**MAE**	**ACC**		
**RF**	9.1	0.83	0.67	0.68		
**GBM**	7.82	0.86	0.65	0.69		
**conv**	5.18	0.86	**0.63**	**0.7**		
**dconv**	4.45	0.88	**0.63**	**0.7**		
**LSTM**	3.71	0.89	**0.63**	**0.7**		
**AllesTM**	**3.72**	**0.9**	**0.63**	**0.7**		
**MEMSAT-SVM**		0.74				
**PolyPhobius**^**a**^		0.75/0.75				
**SCAMPI**^**a**^		0.77/0.78				
**PROFbval**			0.78	0.65		
**PredyFlexy**			0.8			
	**Phi angles**	**Psi angles**	**Secondary structure**	**RSA monomer**	**RSA complex**	**RSA change**
	**MAE**	**MAE**	**ACC**	**MAE**	**MAE**	**MAE**
**RF**	18.91	39.5	0.82	0.18	0.16	0.09
**GBM**	18.5	35.93	0.84	0.17	0.15	0.09
**conv**	16.93	28.75	0.85	0.16	**0.14**	**0.07**
**dconv**	17.43	29.47	0.85	0.16	**0.14**	**0.07**
**LSTM**	17.03	28.81	0.85	0.16	**0.14**	**0.07**
**AllesTM**	17.34	30.41	0.86	0.15	**0.14**	0.08
**PROFphd**			0.78			
**PSIPRED**			0.87			
**ANGLOR**	19.57	38.33				
**SPINE X**	20.68	41.77	0.77	0.2	0.17	
**SPOT-1D**	**15.85**	**23.51**	**0.89**	**0.14**	0.16	

^a^The second value indicates the performance of PolyPhobius and SCAMPI without reentrant regions

Again, all our models perform better on the independent test dataset by a wide margin. In particular, AllesTM, our final method, achieves an ACC of 0.85 during cross-validation and an unprecedented ACC of 0.9 on the independent test dataset. In contrast, MEMSAT-SVM, PolyPhobius and SCAMPI exhibit similar performance on the two datasets (ACC of 0.74, 0.75 and 0.77 on the independent test data set, respectively). Thus, it appears that the datasets have a similar difficulty but the additional ensembling from the cross-validation models boosts the prediction performance even further. With an AUC of 0.7 on RER segments, AllesTM now outperforms MEMSAT-SVM (AUC of 0.58) by a wide margin also on the independent test dataset.

### Flexibility

B-factors were normalized for each protein and predicted as continuous values as well as in terms of two classes, i.e. flexible and non-flexible. In contrast to the z-coordinate and topology predictions a large receptive field seems to be of less importance for the prediction of continuous and two-state flexibility. For example, comparing the performance of the GBM and the LSTM on the independent test dataset the MAE is only 0.02 smaller for continuous flexibility (0.65 and 0.63 respectively) and the AUC is only better by 0.01 for two-state flexibility (0.65 to 0.66 respectively) (Table 1, S3 and S4 Tables, as well as Fig. S5, Fig. S6, Fig. S7 and Fig. S8). In line with these observations, the blending approach, i.e. combining different models in the final method AllesTM, results in only a small performance improvement. AllesTM achieves an MAE of 0.51 for continuous flexibility on the independent test dataset, compared to an MAE of 0.4 for PROFbval and 0.13 measured for PredyFlexy. While the Neural Network (NN)-based PROFbval performs well compared to our RF model it is not specifically trained using TMPs and was trained on a substantially older dataset. While the creators of PredyFlexy claim a correlation with the target variable of 0.71, we were not able to reproduce this performance. As PredyFlexy predicts flexibility by mapping structure fragments with known flexibility values to the sequence, we presume that no TMPs were included in its fragment library.

Regarding the performance of two-state flexibility predictions a similar picture emerges. AllesTM achieves an AUC of 0.66 on the independent test dataset, 0.1 better than PROFbval. The difference in performance during cross-validation and on the independent test dataset is only marginal for the continuous as well as the two-state flexibility. As an example for continuous flexibility, AllesTM has an MAE of 0.66 on the cross-validation dataset but only 0.63 on the independent test dataset. PROFbval, on the other hand, performs slightly worse on the independent test dataset compared to the cross-validation dataset (MAEs of 0.78 and 0.77 respectively).

### Torsion angles

AllesTM only predicts the φ and ψ angles because the third dihedral angle, ω, is essentially fixed at 180°. Similar to the protein flexibility, long range interactions seem to play a minor role when predicting φ and ψ, as the methods with a larger receptive field only perform slightly better. Therefore ANGLOR, which uses a NN for φ and a SVN for ψ angles, and SPINE X which uses a multi-layer NN, perform slightly worse compared to AllesTM (Table 1, S5 and S6 Tables, as well as Fig. S9, Fig. S10, Fig. S11 and Fig. S12). For φ angles in the independent test dataset, SPINE X and ANGLOR achieve an MAE of 20.68 and 19.57 respectively, while the error of AllesTM is 17.34. For comparison, the MAE of our LSTM solution is only 0.31 higher (17.03) while the MAE of our worst performing approach, the RF, is only 1.57 higher (18.91) than that of AllesTM, and still ahead of SPINE X and ANGLOR. Nevertheless, looking at the MAE, SPOT-1D outperforms our method AllesTM (15.85 and 17.34), but with a slightly higher RMSE (31.03 and 31.69). That means that AllesTM, while being on average less accurate, makes fewer large mistakes.

The results are similar for ψ angles, although SPINE X achieves a slightly lower MAE than our RF method on both, the cross-validation (MAEs of 39.76 and 42.91, respectively) as well as the independent test dataset (MAEs of 38.33 and 39.5, respectively). In terms of RMSE, SPINE X trails behind the RF model for the ψ angles on both datasets. While performing with a similar MAE, ANGLOR achieves a much better performance according to the RMSE compared to SPINE X on the independent test dataset (57.88 and 77.41, respectively). In this case SPOT-1D with an MAE of 23.51 and a RMSE of 50.11 outperforms AllesTM with an MAE of 30.41 and a RMSE of 50.73. Both methods, AllesTM and SPOT-1D use LSTMs with their large receptive field, and the previous comparisons suggest that the size of the receptive field only has a minor impact on φ and ψ angle prediction performance, so the strength of SPOT-1D has to lie elsewhere. The two main differences are that SPOT-1D uses predicted contacts as a feature and a larger training dataset, as it is not restricted to TMPs. Therefore there are three possible explanations why contacts bring this benefit. The first is that contacts of residues which are sequentially very far apart such that even the receptive field cannot incorporate them have an impact on the prediction target. An alternative explanation is that the corellated mutations of sequentially co-located residues provide a very strong signal. The third possible explanation is that the usage of a larger trainging dataset outweights the specifics of TMPs.

### Secondary structure

Table 1 and S7 Table show the performance of AllesTM with its underlying models, SPINE X, PROFphd, PSIPRED and SPOT-1D for the three-state prediction of secondary structure, i.e. α-helix, β-strand, and coil. Looking at the overall accuracy (ACC) on the cross-validation as well as on the independent test dataset, AllesTM, PSIPRED and SPOT-1D perform best: AllesTM with an ACC of 0.84 and 0.86 respectively for both datasets and PSIPRED with an even higher ACC of 0.85 and 0.87 and SPOT-1D with a remarkable ACC of 0.88 and 0.89. Other models closely follow, with RF being the worst performer (ACC of 0.8 and 0.82 on the two datasets). SPINE X and PROFphd are slightly behind, with an ACC of 0.77 and 0.78 on the independent test dataset, respectively. With the overall ACC being rather close especially for our developed models, it could be assumed that the size of the receptive field does not play a significant role for the prediction of secondary structure. Zooming into the performance of the methods for α-helix, β-strand and coil separately reveals that this is only true for α-helices and coils. Using the AUC as a threshold-independent measure, the performance for predicting helices correlates almost perfectly with the overall ACC (Fig. S13 and Fig. S14). The RF model, for instance, with its ACC of 0.82 on the independent test dataset has an AUC of 0.8 on helices, while the LSTM achieves an ACC of 0.85 and an AUC of 0.86 on the same dataset and the same class. For β-strands, however, the situation is different. The AUCs of the RF and the GBM, the two models with the smallest receptive field, are 0.62 and 0.73 on the independent test dataset, respectively. The models with a larger receptive field, including conv, all achieve an AUC of 0.79 and above for β-strands. This is also in line with the observation that SPOT-1D especially excels at predicting β-strands (AUC of 0.88), presumably by combining an LSTM, which has a large receptive field, with contact predictions as an input feature.

Furthermore, the exceptional performance of PSIPRED and SPOT-1D leads to the conclusion that secondary structure in TMPs can be predicted without data or algorithms specifically tailored to that protein class. In order to fortify this statement, we evaluated the performance of all prediction methods only on the residues not located in the membrane (S8 Table). The ACC of AllesTM as well as PSIPRED and SPOT-1D (and all other methods) suffers from excluding the TM segments. As an example, the ACC of AllesTM drops from 0.78 to 0.73 on the independent test dataset. This indicates that these regions, which are mostly helical, are relatively easy to predict due to their highly hydrophobic nature and degenerate amino acid composition. Additionally, this comparison shows that for some cases including data from globular proteins is beneficial for the prediction performance, be it because of the larger amount of training data, or because the differences between transmembrane and globular proteins are not as relevant for this specific target.

### Relative solvent accessibility

We predicted the relative solvent accessibility (RSA) for each residue in three forms: i) the monomer RSA, which is the RSA of a residue if the protein chain is not bound to any other chain, ii) the complex RSA, which is the RSA of a residue taking the whole complex into account, and iii) the change of RSA upon complex formation, i.e. if a residue is part of the interaction interface.

**Fig. 2:**
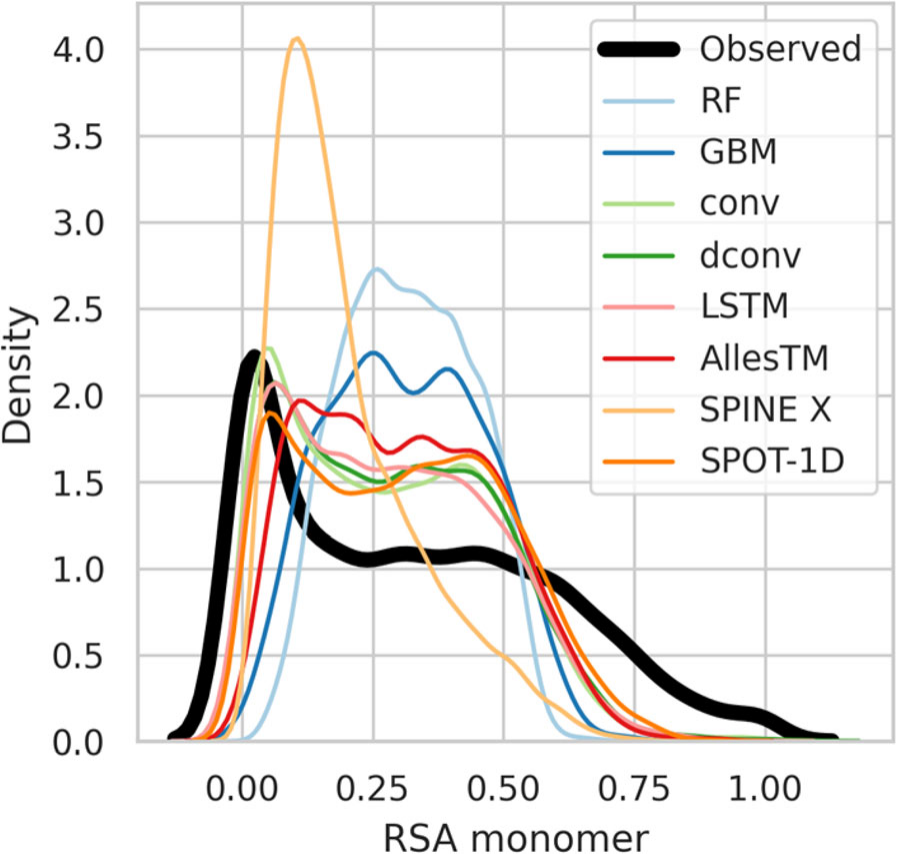
Distribution of the observed and predicted monomer solvent accessibility values in the independent test dataset

Table 1, S9, S10, and S11 Tables as well as Fig. S15, Fig. S16, Fig. S17, Fig. S18, Fig. S19 and Fig. S20 show the performance of our models including the final method AllesTM for these three prediction targets. The prediction performance of AllesTM for the monomer and the complex RSA is very similar. For example, AllesTM achieves an MAE of 0.15 and 0.14 on the independent test data, respectively, which is representative for the other models as well. Long range signals, which would be captured by a larger receptive field, have a noticeable impact on the MAE (e.g. 0.18 for RF and 0.15 for AllesTM on the independent test dataset predicting the monomer RSA), but not on the RMSE (e.g. 0.22 and 0.2, respectively). As the RMSE, which is similar for all models, is sensitive especially to large prediction errors, the difference in prediction performance for the RSA cases seems to be rather nuanced and only impacting the MAE. For the monomer and complex RSA benchmarks, we included SPINE X and SPOT-1D as well. SPINE X lags behind AllesTM independent of the applied performance measure but performs better on the complex RSA (MAE of 0.17) than on the monomer RSA (MAE of 0.2). For SPOT-1D it is the other way around as it even outperfoms AllesTM slightly on the monomeric RSA with an MAE of 0.14 compared to 0.15 of AllesTM, while lagging behind on the complex RSA (MAE 0.16 compared to 0.14, respectively). According to the benefits of using contact predictions as input features for targets such as the secondary structure, it is surprising that this feature seems to have a rather small impact on RSA prediction. Figure 2 supports the previous observations showing the distribution of the actual monomer RSA values and the predicted ones. Although not explicitly visible from the numbers, the distribution of RF and GBM models’ predictions is clearly of different shape than the observed distribution.

Compared to the other targets, the prediction of RSA changes does not show a very clear picture, or at least is different from the previous observations and changes dependent on the measure applied. Regarding the RMSE, for example, the RF and GBM models are on par with the final AllesTM method, achieving 0.13 on the cross-validation data and 0.15 on the independent test data outperforming the conv, dconv and LSTM models. For MAE, however, the opposite is the case as AllesTM, RF and GBM do not achieve an MAE as low as the conv, dconv and LSTM models.

## Discussion

In contrast to most of the previously proposed methods, usually focused on just one property, AllesTM predicts 10 different structural features of transmembrane proteins, i.e. almost every aspect of protein structure that can be extracted from atomic coordinates. It blends several state of the art machine learning algorithms: random forests and gradient boosting machines, convolutional neural networks in their original form as well as enhanced by dilated convolutions and residual connections, and, finally, long short-term memory architectures. All predictions were carefully evaluated by 5-fold cross-validation, tested on an independent dataset, and compared to the respective state of the art methods.

We found that the size of the receptive field, i.e. the number of adjacent residues considered while making predictions for a specific residue position, has a varying impact for different prediction targets. This is also true for contact predictions which are used by SPOT-1D as an input feature, giving the tool an advantage for some targets. Furthermore, using a method specifically geared towards TMPs is not always beneficial or at least can be compensated by the availability of more training data, as is for example the case for secondary structure prediction accuracy.

## Conclusions

In terms of prediction accuracy, the main results can be summarized as follows:

- **Z-coordinate**. Being the only publicly available method for this particular target, AllesTM predicts Z-coordinates for individual residues with an average error of 3.72 Å (about 12% of the average membrane thickness), therefore locating the residues reasonably well.
- **Topology**. AllesTM achieves an accuracy of 0.9 in a four-state prediction (inside, outside, transmembrane, and re-entrant regions) and thus outperforms the leading methods MEMSAT-SVM, SCAMPI and PolyPhobius.
- **B-factors**, i.e. residue flexibility. AllesTM achieves an MAE of 0.63 for continuous value predictions and an AUC of 0.66 for two state (flexible/non-flexible) predictions. The respective values for PROFbval are 0.78 and 0.56.
- **Three-state secondary structure**. With an accuracy of 0.86 AllesTM lags slightly behind PSIPRED (0.87) and SPOT-1D (0.89).
- **Relative solvent accessibility** (RSA). For proteins complexed with other chains (MAE of 0.14) AllesTM outperforms SPINE X by approximately 20% and SPOT-1D by a small margin (MAE of 0.16). For monomers (MAE of 0.15) SPOT-1D is marginally better by an MAE difference of 0.01. The difference between monomers and their bound forms is unique to AllesTM and can be predicted with an MAE of 0.08.
- **Torsion angles φ and ψ**. AllesTM (MAEs of 17.34 and 30.41 for φ and ψ, respectively) performs better than ANGLOR (19.57 and 38.33) but worse than SPOT-1D (15.85 and 23.51).

By providing the multitude of prediciton targets and avoiding the use of complex dependencies characteristic for many other prediction tools, AllesTM is easy to setup and run, making it a useful universal tool for structural bioinformatics studies. AllesTM was developed using Python and is available as a standalone tool having only HHblits as a non-Python dependency. It can be either installed from GitHub or via the Python Package Manager. See https://github.com/phngs/allestm for detailed instructions.

## Materials and methods

### Dataset

We retrieved from the Orientations of Proteins in the Membranes database (OPM) [10] all 4357 entries belonging to either the “Alpha-helical polytopic” or the “Bitopic proteins” class, each containing one or multiple protein chains with known 3D structures. OPM entries contain information about the thickness of the membrane and the relative position of the protein with respect to it. In addition, OPM offers modified PDB (Berman et al., 2000) files, with proteins rotated and translated in such a way that each atom’s z-coordinate corresponds to the depth of that atom in the membrane. A z-coordinate of 0 means that the atom is located at the center of the membrane, while values deviating from 0 indicate the distance of an atom from the membrane center measured in Å. These distances can be either negative or positive, depending on which side of the membrane the atom is located at. Positive z-coordinates correspond to the side of the membrane facing the compartment, which is more “outside” compared to the other side of the membrane, where z-coordinate values are negative. For example, z-values for the inner and outer bacterial membranes are assigned in such a way that the periplasmic space is annotated with positive values for the inner membrane (negative values represent the cytoplasm), and negative values for the outer membrane (where positive values represent the extracellular space). If the absolute value of a Cα atom’s z-coordinate exceeds a half of the membrane’s thickness (which is about 15.1 ± 1.0 Å in our final dataset according to the OPM database), the residue is considered to be located outside of the membrane. Protein chains not crossing the membrane at least once, i.e. not having at least one Cα atom on both sides of the membrane, were excluded from consideration.

We only considered protein structures solved by X-ray crystallography with a resolution of 3.5 Å or better. Protein chains shorter than 30 residues as well as those containing only one amino acid type were ignored. Because some OPM entries had no properly annotated B-factors, we required structures to have more than one different B-factor value. Amino acid sequences were extracted from the ATOM records of the PDB entries (atomseq), and only proteins with coordinate information available for the backbone atoms N, C and Cα of every amino acid were retained for further analysis. Because the atomseq records do not always correspond to the complete protein sequence provided in the seqres records, we required at least 80% of the seqres sequence to be covered by the atomseq sequence.

Missing residues can result in unrealistically large steps between the coordinate, B-factor torsion angle and solvent accessibility values if they are consecutive according to the atomseq sequence but not to the seqres sequence. For this reason, we only allowed missing residues at the beginning and the end of the atomseq sequence but did not consider them during training and benchmarking of the classifier. These filtering steps resulted in a dataset of 5375 high-quality protein chains, which were subjected to redundancy reduction and cross-validation described in the next section.

### Redundancy reduction and cross-validation

**Fig. 3:**
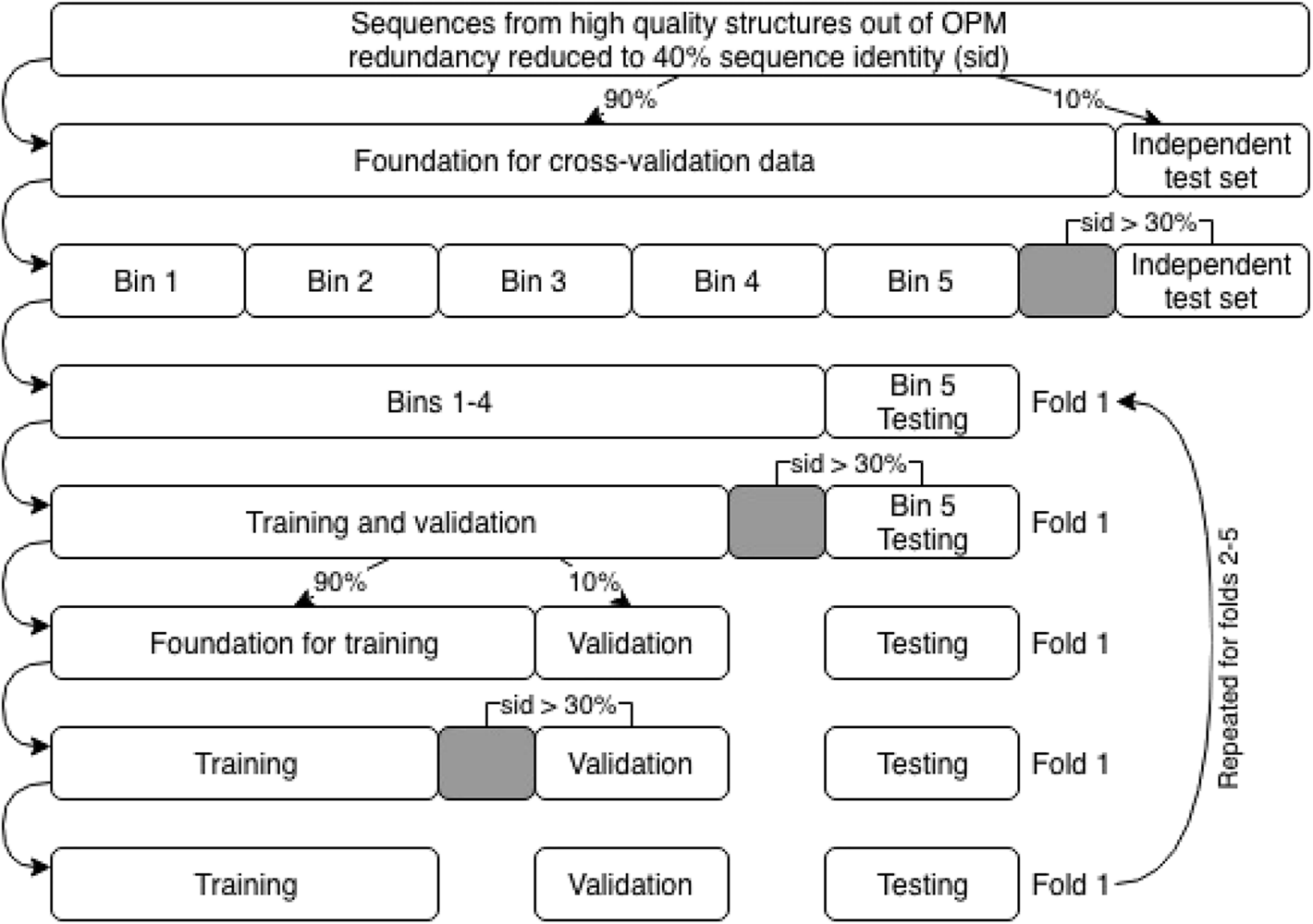
Datasets used for 5-fold cross-validation and testing. Gray boxes indicate sequences excluded from consideration as they shared more than 30% identity with the test data. See text for further details

While it is desirable to retain as many sequences as possible for training a machine learning model, there are some practical limits to the amount of data that can be used. First, the training process can be quite computationally intensive depending on the algorithm used, and second, redundant data leads to an overestimation of the method’s performance. Therefore, we chose to combine a two-step redundancy reduction procedure with a standard 5-fold cross-validation and testing on an independent dataset (Fig. 3). First, the initial dataset containing the 5375 protein chains was made non-redundant at the 40% sequence identity level using CD-HIT [11], which resulted in a much smaller dataset of only 302 sequences well suitable for computer-intensive training procedures. Thirty-one of these sequences, i.e. 10%, were set aside as independent test data and not employed for any training or parameter tuning until the final models were built. The remaining 271 proteins, or 90%, formed the foundation for the cross-validation data. In order to provide a fair assessment of our methodology, we ensured an equal distribution of protein topologies (proteins with a given number of transmembrane segments (TMS)) in both parts of the split. From the initial cross-validation data, all proteins which had a sequence identity above 30% to any protein in the independent test data were removed, resulting in 178 proteins. These sequences were randomly split into five equally sized bins, with four of these bins used for training and validation, and one bin for testing, again ensuring equal distribution of protein topologies. In order to not overestimate the performance of our predictor during cross-validation, we removed all proteins from these five bins that shared a sequence identity greater than 30% with any protein in the testing bin. This approach guarantees that the test bin is completely independent from the training and validation bins, while retaining as many proteins for training as possible. In order to be able to choose the hyperparameters of the learning algorithms (i.e. the parameters, which affect the model but have to be chosen prior to the training process) without using the test bin or even the independent test set, the 4 training and validation bins were split further. 90% of the sequences were used to learn the model’s parameters (training), while 10% were used to estimate the best performing hyperparameters (validation). Similar to the process employed in the previous splits, topologies were equally distributed and proteins in the training dataset sharing more than 30% sequence identity to any protein in the validation dataset were removed. We performed these steps 5 times using each bin, and therefore each protein, for testing exactly once. Because of the rigorous redundancy reduction inside each fold, the distribution by protein topology resulted in the folds being of slightly different sizes (S12 Table).

### Prediction targets

#### Residue z-coordinates and protein topology

The depth of a residue in the membrane was obtained from the z-coordinate of its Cα atom in the modified PDB files derived from the OPM. Because we are mainly interested in the z-coordinates of residues inside and adjacent to the membrane boundaries, we limited the z-coordinate values to the range from − 25 to 25 Å as the membrane thickness normally does not exceed 30 Å. Values outside that range were set to − 25 and 25 Å, respectively. For training and prediction we scaled the z-coordinate values to a range from − 1 to 1, while for reporting the results the actual values were used.

In order to assign residues to discrete states, we determined four types of topological domains for each transmembrane protein. Initially, TMSs were defined as segments of which both ends have a distance of at most 10 Å to the opposite sides of the membrane. These segments are extended in both directions as long as they touch the membrane boundaries. Next, reentrant regions (RERs) were identified by finding stretches of amino acids which enter and exit the membrane on the same side, consist of at least 3 amino acids and immerge at least 3 Å into the membrane [12]. Finally, the remaining segments, i.e. those which are neither TMSs nor RERs, were annotated as domains residing on either the inner or the outer side of the membrane depending on whether their residues’ z-coordinates were negative or positive, respectively. All residues were assigned to one of the four discrete states - inside, TMS, outside and RER – and encoded by binary vectors [1, 0, 0, 0], [0, 1, 0, 0], [0, 0, 1, 0] and [0, 0, 0, 1], respectively.

#### Continuous and two-state flexibility

Residue flexibility was represented both as a continuous and a two-state discrete variable based on the B-factors derived from the PDB entries. Because B-factors are heavily dependent on the experimental conditions and the structure resolution, they are not directly comparable between different structures. We therefore converted them into z-scores for each protein separately using the formula

$${B}_{norm}=\frac{B_{\mathrm{r} aw}-\mu }{\sigma }$$

where B_raw_ is the original B-factor of a residue’s Cα atom, μ and σ are the mean and the standard deviation of all B-factors for a particular protein, respectively, and B_norm_ is the continuous residue flexibility used as a prediction target. Continuous flexibility was converted into two discrete states for flexible (Bnorm > 0.03) and rigid (Bnorm ≤0.03) [13], numerically represented as 1 and 0, respectively.

A recent study suggested an approach to estimate the maximal average values of B-factors at a given crystallographic resolution [14]. We found that in roughly a half of the structures in our independent dataset B-factors actually exceed the proposed maximal values, presumably as a consequence of the membrane proteins being removed from their natural stabilizing environment. Given the small number of available structures we chose not to exclude from consideration the atomic records with excessive B-factor values, but this issue definitely deserves further consideration.

#### Torsion angles

The conformation of the protein backbone can be characterized using the three dihedral angles, which are the rotations around the bonds between N and Cα, Cα and C, C and N, respectively. The values of these angles are continuous and range from − 180° to 180°. Because the torsion angle ω is essentially flat and fixed to 180° due to the partial double-bond character of the peptide bond, we are only predicting the φ and ψ angles. The values of torsion angles were converted to the range from − 1 to 1.

### Secondary structure

Secondary structure assignments were calculated from known 3D structures using DSSP [15, 16]. While DSSP defines the total of eight different secondary structure states, we followed the common approach and collapsed them to three states. H (α-helix), G (3_10_-helix) and I (π-helix) were converted to helix, E (extended strand) and B (isolated β-bridge) to β-sheet, and all the remaining states - T (turn), S (bend) and coil, to coil. These three states were presented to the learning algorithms as binary vectors: [1, 0, 0], [0, 1, 0], [0, 0, 1] for helix, β-sheet and coil respectively.

#### Relative solvent accessibility

The DSSP program was also utilized to calculate three continuous targets for each residue concerning relative solvent accessibility (RSA). The absolute solvent accessibility of each residue, expressed in Å^2^, was normalized by dividing it by the maximum solvent accessibility according to [17]. The first RSA target was calculated taking into account only the amino acid chain to which a given residue belongs, i.e. treating the protein as a monomer. The second RSA target was calculated taking into account all protein subunits, while the third target was defined as the difference between both values and therefore representing the change of RSA upon complex formation.

### Input features

We used the amino acid sequence and the evolutionary profile of the query protein as input to the classifier. The amino acid sequence was presented to the model using the standard one-hot encoding. A residue is represented by a vector of length 20 initialized with zeros, in which each position corresponds to one of the 20 standard amino acids. Only the position which corresponds to the current amino acid is set to one. The entire protein sequence is thus represented by a *Lx20* matrix, where *L* is the length of the sequence.

An evolutionary profile is another *Lx20* matrix representation of the input protein sequence derived from a multiple sequence alignment (MSA). Each value of the matrix indicates to which extent a specific amino acid is over- or underrepresented at a specific position of the alignment. More precisely, each value m_i,j_ of the *Lx20* matrix is the log ratio of the relative frequency *p*_*i,j*_ of the amino acid *i* at column *j* in the MSA and the relative frequency *p*_*i*_ of the amino acid *i* in the entire alignment, normalized with the sigmoid function:

$${m}_{i,j}=\frac{1}{1+\exp \left(-\log \left({p}_{i,j}/{p}_i\right)\right)}$$

If the amino acid *i* does not occur in the entire alignment or at a given position *j* of the MSA, the respective values of *p*_*i*_ or *p*_*i,j*_ can become zero, rendering the logarithm of *p*_*i,j*_*/p*_*i*_ invalid. To overcome this issue, we added a pseudo count of one to each *p*_*i,j*_ and a pseudo count equal to the number of columns in the alignment to each *p*_*i*_.

MSAs were calculated from top-scoring hits obtained by searching the Uniclust30 database [18] using HHblits, an iterative hidden Markov model-based method [19], with all default parameters.

### Prediction algorithms

#### Neural network architecture

**Fig. 4:**
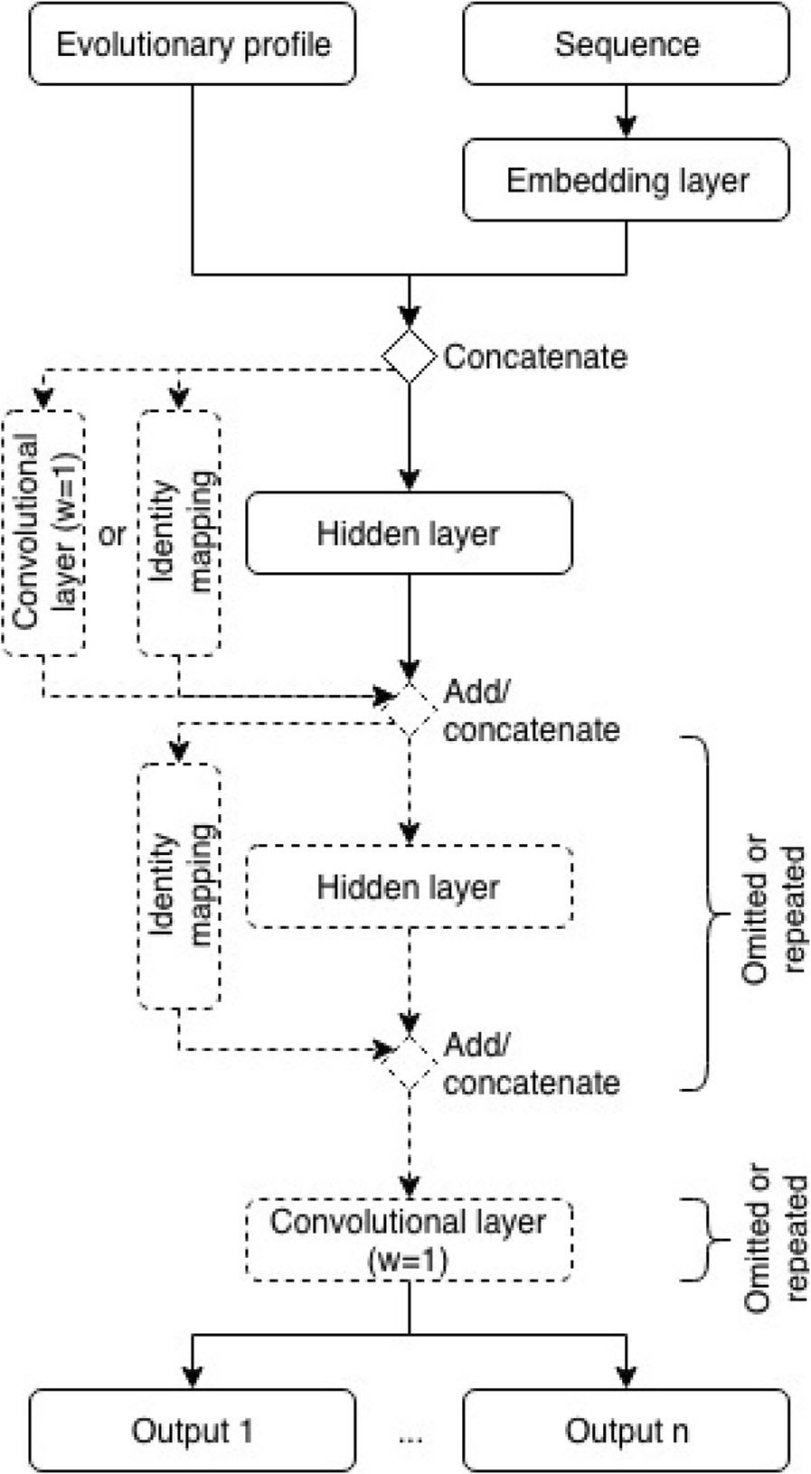
Overview of the neural network architecture. The embedding layer processes the sequence feature before its output is concatenated with the evolutionary profile feature and passed to the hidden layers. As described in the text each hidden layer is either a convolutional layer, an LSTM layer or a dilated convolution block, and the number of such layers is determined by hyperparameter selection. Another hyperparameter is whether an identity mapping is used or not. If this is the case, the input to each hidden layer additionally bypasses that layer to be added or concatenated to its output. Feeding the next layer with both the input as well as the output of the previous layer enables it to correct errors introduced by the previous layer. In case the first hidden layer’s inputs are added to its outputs instead of concatenating them, their dimensions must be aligned to make this operation possible. Therefore, the identity mapping is realized by another convolutional layer with a widow size of one in order to allow for the addition operation. After the hidden layers, several additional convolutional layers may be used with a window size of one, which are connected to the output neurons representing the actual predictions

We implemented three different NNs sharing a similar basic architecture consisting of three building blocks (Fig. 4). In the following, the differences and the similarities between these NNs as well as the hyperparameters are described in more detail.

The input and embedding layer form the entry point where the sequence and the evolutionary profile are fed to the NN. Before the two *Lx20* matrices representing these features are combined, the sequence feature is processed by a so-called embedding layer [20]. During the training process, this layer learns to map the vectors of length 20 containing the binary encodings of the amino acids to a vector of *n*_*embedding*_ real numbers, with each value of the vector potentially representing an amino acid property. The rationale behind this mapping is that the vectors of two amino acids with similar properties are closer to each other in terms of the Euclidean distance between them than two amino acids with different properties. The benefit of this approach is that residue-related features such as physicochemical properties do not have to be manually selected but are rather inferred from the data. The application of the embedding layer to the sequence feature matrix results in a matrix of dimension Lxnembedding, which is then combined with the evolutionary profile to form a *Lx (20 + n*_*embedding*_*)* sized matrix to be fed to the hidden layers.

The hidden layers are the variable building blocks between the three NN architectures. They either consist of *n*_*hidden_layers*_ standard convolutional layers (conv layers), blocks of dilated convolutional layers (dconv blocks) or layers containing Long Short-Term Memory cells (LSTM layers) [21, 22].

Conv layers are equivalent to the established sliding window approach, i.e. the same set of *n*_*hidden_neurons*_ neurons is applied to sequence segments in each position, with the central residue serving as a prediction target. The number of residues in the segment is defined by the window length *w*, which is also commonly known as kernel size in the deep learning literature.

Dconv blocks contain at least two layers equivalent to conv layers, except that the residues in the window are not directly adjacent. Instead they are spaced according to the dilation rate d. Considering the central residue at position *i*, a window size of 5 and a dilation rate d of 2, the complete window would contain the residues *i-4, i-2, i, i + 2, i + 4*. The benefit of this approach is the expanded receptive field. Given a specific residue for which a target is predicted, the receptive field defines how many adjacent residues are considered when doing the prediction. By increasing the dilation rate exponentially for each subsequent hidden layer, the receptive field also increases exponentially while the number of free parameters only increases linearly. Each dconv block contains n_dconv_layers_ layers, resulting in a total number of *n*_*hidden* _ *layers*_ ∗ *n*_*dconv* _ *layers*_ hidden layers. The dilation rate *d* increases with each subsequent layer *l* in a dconv block according to the formula *d*_*l*_ = 2^*l* − 1^. The conv as well as the dconv layers use the rectified linear unit (ReLu) activation function, which has replaced the sigmoid function as the most commonly used activation function in NNs [23]. The output of a ReLu is calculated as max(0, *x*), with *x* being the input to the ReLu activation function. One of ReLu’s benefits is that it does not get saturated even for large input values, mitigating the problem of vanishing gradients which can occur in deep NNs when using the sigmoid or tanh activation functions.

**Fig. 5:**
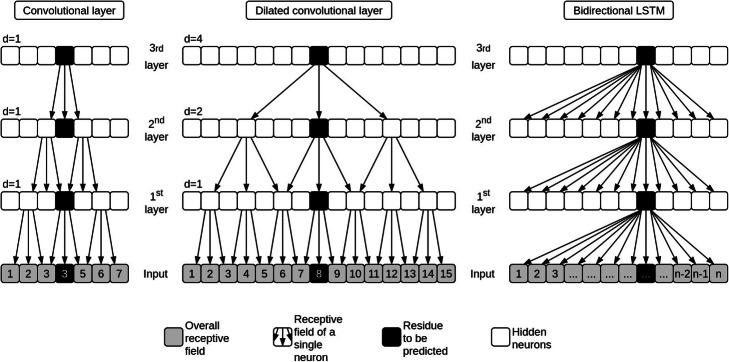
Impact of different types of hidden layers on the receptive field. The receptive field reflects the number of adjacent residues considered when making a prediction for a specific residue. Using a window size of three, the classic neural network architecture consisting of convolutional layers achieves a receptive field of size seven after three hidden layers. Using an increasing dilation rate for each layer, the receptive field can be expanded to 15 residues using the same window size and number of layers. For a bidirectional LSTM, the information from the complete sequence is available at each position and each layer

LSTM layers are a type of recurrent neural network layers (RNNs) widely applied in the field of speech recognition and text analysis. Unlike the conv layers, whose receptive field is limited by the filter or window size, RNNs are capable of processing the entire sequence. One can imagine a RNN as a filter of size one which gets applied to one sequence position after the other. The difference to a convolutional layer is that instead of being independent of the previous position, the LSTM has access to the previous position’s output which gets combined with the input of the actual residue. In our model, we are using LSTMs in a bidirectional way by applying two separate LSTMs, one on the actual and one on the reversed sequence. For each sequence position the outputs of each of the two LSTMs are processed by a tanh activation function and concatenated to serve as input for the next layer in the network. Figure 5 visualizes the difference between the receptive fields for different types of hidden layers and a specific set of hyperparameters.

An additional hyperparameter is the use of residual connections, also called skip connections. This strategy allows a given hidden layer to learn from the mistakes of the previous hidden layer. This is achieved by creating a connection, called identity mapping, which combines the output of the previous hidden layer with the output of the actual layer, essentially bypassing it. This combination is performed by either adding the respective outputs of the previous hidden layer and the actual layer or concatenating them. A special case is the identity mapping used with the first hidden layer in case addition is used as the combination function. Because the dimensions of the input, i.e. a *Lx (20 + n*_*embedding*_*)* sized matrix, does not match the output dimensions of the first hidden layer, their elements cannot be added. Therefore, a conv layer with a window size of one and a number of neurons equivalent to the number of outputs from the first hidden layer is used as the identity mapping.

Finally, the dense and output layers transform the results from the hidden layers into the desired output. The dense layers are a set of *n*_*dense_layers*_ conv layers with a window size of one and a number of *n*_*dense_neurons*_ neurons using the ReLu activation function. Their result is processed by the output layers, each directly connected to the last dense layer. The output layers are again conv layers with a window size of one but each with the number of neurons matching the prediction target dimensionality, i.e. one for the continuous targets such as z-coordinates or angles, one for the two-state flexibility as it is represented as a binary value, and three or more for categorical targets such as secondary structure or topology. The activation function is also dependent on the prediction target: linear activations for continuous targets, binary cross-entropy for binary targets, and categorical cross-entropy for categorical targets.

#### Neural network training

While the previously mentioned hyperparameters affect the architecture of the NNs, there are a few more decisions to be made in order to optimize the training process: the batch size, the optimization algorithm and its learning rate, and the method for optimizing the hyperparameters. A batch is a number of training examples which are presented to the NN and the optimization algorithm before the weights of the model get updated. It therefore leads to an update of the weights depending not only on the error on a single sample, but also on the average error over a specific number of samples. The optimization algorithm is used to update the weights of the neurons in the NN while the learning rate determines how much these weights are adapted after each batch. As designing and training a state of the art NN is often described more an art than an exact science, we ran some initial tests resulting in the following decisions. By trial and error, we settled on Adam as the optimization algorithm of our choice as it leads to the fastest convergence and therefore reduced training time for each experiment [24]. In contrast to a global learning rate, the Adam algorithm maintains a learning rate for each trainable weight in the network and adapts the weights based on the recent magnitudes of their gradients. Furthermore, we narrowed down the number of hyperparameter values describing the architecture to the most promising candidates with respect to prediction performance and runtime based on manual trials (S13 Table).

Because a grid search, i.e. testing all possible parameter combinations, would be computationally infeasible, we employed further strategies to make the parameter search and the training more efficient. Similar prediction targets were combined into one NN containing the output neurons for multiple prediction targets, thereby reducing the number of NNs to be trained. We conducted a trial-and-error assessment of the impact exerted on model performance by specific combinations of prediction targets compared to training the models separately. As a result, we combined the z-coordinates with the topology, the continuous with the two-state flexibility, the two torsion angles, and the three solvent accessibility targets. Only secondary structure was predicted most accurately without the influence of other targets. This results in only five NNs per type of hidden layer/block, i.e. only 15 different NNs. Instead of an exhaustive grid search, we used a random parameter search with the possible values given in S13 Table. More precisely, for each type of NN and for each of the five folds a random set of the possible values was chosen. If the chosen set of parameters performed better for a certain type of NN and fold combination it was accepted as the currently best solution. Two additional measures were used to further speed up the training process: early stopping and reducing the learning rate during training. Early stopping is a procedure to abort the training process if no further improvements are expected. This is achieved by evaluating the current NN on the validation set after one iteration through all training samples. If the performance on the validation set stagnates or even decreases, the training is stopped early. Because performance fluctuations are quite normal, the training proceeds for up to 10 more iterations after the currently best result has been achieved, unless another performance increase occurs. If the training is stopped, the state of the model at the iteration with the highest performance is selected as the final model. The early stopping approach has two benefits: it prevents overfitting and unnecessary iterations which are computationally intensive and time-consuming. While after 10 iterations the training stops completely, the learning rate is multiplied by 0.1 and therefore reduced automatically after 5 iterations without performance increase. This approach allows for faster convergence by using a higher learning rate in the beginning of the training and more precise weight updates (albeit with a lower learning rate) at the end.

The neural networks were implemented using the deep learning framework Keras [25], which is based on the TensorFlow library [26].

#### Random forest

In addition to the three types of NNs, a random forest (RF) was implemented using the scikit-learn package [27, 28]. A random forest is an ensemble method that employs *n*_*trees*_ decision trees. The rationale for using this approach is to combine many classifiers/regressors, which are themselves as accurate as possible, while being diverse at the same time. To achieve this, a subset of training samples is created for each tree using bootstrapping. Additional diversity is introduced by using only the fraction *n*_*features*_ of all input features for each tree. A decision tree is trained on a randomly selected subset of training examples and features and then choosing the feature, which provides the best split between the classes to be used for the first decision. This process is then performed for the two resulting subtrees, their subtrees and so forth. The default criteria used for the splits are mean squared error for regression problems and Gini impurity for classification problems. The output of the random forest is either a value between 0 and 1 for regression and binary classification problems, and a vector of values between 0 and 1 with the length equal to the number of output classes for multiclass classification problems. Significant benefits of the random forest approach are that it does not require data normalization, is not susceptible to overfitting, and reveals the importance of each feature and its impact on the prediction. In addition to *n*_*trees*_ and *n*_*features*_ two further parameters were optimized: the minimum number of samples in each tree’s leaves *n*_*min_samples*_, which effectively reduces the depth of the trees, and the window size *w*. Because training an individual random forest model is fast, we were able to do a complete grid search over the parameters *n*_*features*_, *n*_*min_samples*_, and *w* (S14 Table), while *ntrees* was determined by repeatedly adding 100 trees until the performance on the validation data did not increase further.

#### Gradient boosting machine

The fifth algorithm used is a gradient boosting machine (GBM) implemented in the XGBoost package, which can be seen as an enhancement of RFs [29–31]. The difference is that in RFs the trees are independent of each other and can therefore be created in parallel. In GBMs every tree is trained to reduce the error of the preceding tree and therefore they are trained sequentially (which does not mean GBMs cannot use parallel processing). This effectively results in the model focusing more on the difficult samples for which the error is large. More precisely, the subsequent trees are trained on the gradient of a given loss function with respect to the predicted values of the previous tree. The output dimensions and value ranges are equal to those of RFs. Because of the more sophisticated approach and training methodology involving a gradient descent method, GBMs are generally more accurate and require fewer trees until convergence, i.e. until the point where additional trees do not lead to a performance increase. On the downside, they are more sensitive to overfitting and thus require regularization methods, which are controlled by additional hyperparameters. The parameters *n*_*trees*_, *n*_*features*_ and *w* are similar to those of RFs with *n*_*trees*_ again being optimized by early stopping. The additional parameters control the number of samples *n*_*samples*_ used in each tree, the learning rate *r* for gradient descent, the maximum depth *n*_*depth*_ of each tree and the two parameters γ and *n*_*min_child_weight*_, which are used for regularization. Because numerous tunable hyperparameter combinations would have been impossible to test exhaustively, we used a random search approach with the values shown in S15 Table.

#### Blending

Our final predictor, AllesTM, is a combination of the previously described models derived during cross-validation. Similar to the idea to combine multiple weak classifiers on which RFs are based, we combined the tree different NNs with the RF and the GBM for each prediction target using a meta classifier, a process often referred to as blending. Because of the diversity of the classifiers, each of them is able to infer different associations between the input features and the target variable. To combine the methods, we first made predictions for the validation bins in each fold with every classifier created using the training data of the corresponding fold. These predictions were then used as input to train the meta classifier. The methods of choice for the meta classifiers are linear regression for continuous targets and logistic regression for binary and categorical targets, resembling a weighted average over the base models’ predictions. This approach was implemented using the scikit-learn library. Because the models are trained during cross-validation, there are five trained models for each algorithm. In order to make predictions for the independent test dataset as well as for the creation of the final predictor AllesTM, the outputs of the models are averaged. E.g. to predict the z-coordinates of a residue, five meta models exist, each combining the three different NNs with the RF and the GBM. The result of these five meta models is then averaged to produce the final prediction, which therefore can be seen as a combination of 30 different models, i.e. five different algorithms plus one meta model for each fold.

### Comparison with other methods

Because of the variety of prediction targets covered by AllesTM, we had to compare its predictive power with several individual previously published methods. The only target where no comparison was possible were the z-coordinates. Although a method called ZPRED [32, 33] was published to predict the location of a residue in the membrane, it is not publicly available and, according to the publication, its predictions are restricted to the absolute distance of a residue to the membrane center, i.e. not providing information about the side of the membrane at which the residue is located. ANGLOR [34] predicts dihedral angles by using a NN for the φ angles and a support vector machine (SVM) for the ψ angles. SPINE X [35] is a multi-layer NN-based tool for predicting dihedral angles as well as secondary structure and solvent accessibility. We converted the absolute values of solvent accessibility produced by SPINE X to relative values by dividing them by the maximum solvent accessibility of a given amino acid, the same approach as in AllesTM. PredyFlexy [36] derives continuous B-factor values by assigning short structural fragments from a pre-compiled library to the sequence using a SVM-powered scoring scheme. The B-factors of these fragments are then transferred to the target sequence resulting in the prediction. Another tool predicting B-factor values is PROFbval [13]. The method is trained to emit a 10-state flexibility, which is then converted to a binary and a continuous prediction. PolyPhobius [37] and MEMSAT-SVM [38] both predict transmembrane protein topology. While the former is based on hidden Markov models, the latter uses an SVM. MEMSAT-SVM is especially interesting for comparison because of its ability to predict RERs. We also included SCAMPI [39] as a more recent topology prediction method. SCAMPI is another HMM based method which uses the fact, that the N- and the C-terminal helices are more hydrophobic. Two further well-established methods were used to evaluate the secondary structure prediction performance, PROFphd [40] and PSIPRED [41]. Both are using evolutionary information derived from MSAs and two neural networks, one for the initial prediction and a second one for smoothing. Recently PSIPRED was retrained using more layers in order to improve its prediction performance [42]. Finally, we included SPOT-1D [43], a very recent and versatile method capable of predicting secondary structure, solvent accessibility (which we converted to relative values similar to SPINE X) as well as φ and ψ angles. It uses LSTMs and CNNs and takes the output of several other methods, e.g. contact predictions, to enhance prediction performance.

### Evaluation metrics

In order to benchmark the classifier and to compare its performance to other methods, we calculated several performance measures. For the continuous target variables (z-coordinates, flexibility and torsion angles), we calculated mean absolute error (MAE), mean squared error (MSE), root-mean-square error (RMSE), Pearson correlation coefficient (*r*_*p*_) and Spearman’s rank correlation (*r*_*s*_) using the following equations:

$$MAE=\frac{\sum_{i=1}^n\mid {y}_i-{x}_i\mid }{n}$$

$$MSE=\frac{\sum_{i=1}^n{\left({y}_i-{x}_i\right)}^2}{n}$$

$$RMSE=\sqrt{MSE}$$

$$r=\frac{\sum_{i=1}^n\left({x}_i-{m}_x\right)\ast \left({y}_i-{m}_y\right)}{\sqrt{\sum_{i=1}^n{\left({x}_i-{m}_x\right)}^2\ast {\left({y}_i-{m}_y\right)}^2}}$$

where *y*_*i*_ and *x*_*i*_ are the predicted and observed values, *n* is the number of predictions, and *m*_*x*_ and *m*_*y*_ are the means of all predictions and observed values, respectively.

For the discrete target variables (topology and flexibility) we calculated accuracy (ACC), precision (P), recall (R), F1-score (F1) and Matthews correlation coefficient (MCC) as follows:

$$ACC=\frac{TP}{TP+ TN+ FP+ FN}$$

$$P=\frac{TP}{TP+ FP}$$

$$R=\frac{TP}{TP+ FN}$$

$${F}_1=\frac{2\ast P\ast R}{P+R}$$

$$MCC=\frac{TP\ast TN- FP\ast FN}{\sqrt{\left( TP+ FP\right)\ast \left( TP+ FN\right)\ast \left( TN+ FP\right)\ast \left( TN+ FN\right)}}$$

where TP, TN, FP and FN are the number of true positives, true negatives, false positives, and false negatives, respectively.

## Supplementary information

**Additional file 1: S1 Fig.** Distribution of the observed and predicted z-coordinates on the cross-validation dataset. **S2 Fig.** Distribution of the observed and predicted z-coordinates on the independent test dataset. **S3 Fig.** Precision-recall and ROC curves of the predicted topology on the cross-validation dataset. **S4 Fig.** Precision-recall and ROC curves of the predicted topology on the independent test dataset. **S5 Fig.** Distribution of the observed and predicted continuous flexibility on the cross-validation dataset. **S6 Fig.** Distribution of the observed and predicted continuous flexibility on the independent test dataset. **S7 Fig.** Precision-recall and ROC curve of the predicted two-state flexibility on the cross-validation dataset. **S8 Fig.** Precision-recall and ROC curve of the predicted two-state flexibility on the independent test dataset. **S9 Fig.** Distribution of the observed and predicted φ angles on the cross-validation dataset. **S10 Fig.** Distribution of the observed and predicted φ angles on the independent test dataset. **S11 Fig.** Distribution of the observed and predicted ψ angles on the cross-validation dataset. **S12 Fig.** Distribution of the observed and predicted ψ angles on the independent test dataset. **S13 Fig.** Precision-recall and ROC curves of the predicted secondary structure on the cross-validation dataset. **S14 Fig.** Precision-recall and ROC curves of the predicted secondary structure on the independent test dataset. **S15 Fig.** Distribution of the observed and predicted relative solvent accessibility of monomers on the cross-validation dataset. **S16 Fig.** Distribution of the observed and predicted relative solvent accessibility of monomers on the independent test dataset. **S17 Fig.** Distribution of the observed and predicted relative solvent accessibility of protein chains in complexes on the cross-validation dataset. **S18 Fig.** Distribution of the observed and predicted relative solvent accessibility of protein chains in complexes on the independent test dataset. **S19 Fig.** Distribution of the observed and predicted difference in relative solvent accessibility between the bound and the unbound form of a protein chain on the cross-validation dataset. **S20 Fig.** Distribution of the observed and predicted difference in relative solvent accessibility between the bound and the unbound form of a protein chain on the independent test dataset. **S1 Table.** Performance of several algorithms, including the final method AllesTM, for z-coordinate prediction on the cross-validation and independent test datasets. **S2 Table.** Protein topology prediction performance. **S3 Table.** Continuous flexibility prediction performance. **S4 Table.** Two-state flexibility prediction performance. **S5 Table.** φ angles performance. **S6 Table.** ψ angles performance. **S7 Table.** Secondary structure prediction performance. **S8 Table.** Secondary structure prediction performance excluding residues situated in transmembrane segments. **S9 Table.** Monomer solvent accessibility performance. **S10 Table.** Complex solvent accessibility performance. **S11 Table.** Change of solvent accessibility performance. **S12 Table.** Number of sequences in the training, validation, and test datasets across the five folds. Because of the two-step redundancy reduction procedure, the number of proteins in the validation and training parts are dependent on the test data in the particular fold. **S13 Table.** Parameter values used for the different layer types during random parameter search. **S14 Table.** Parameter values used during grid search of random forest models. **S15 Table.** Parameter values used during random search of GBM models.

## Data Availability

- The software AllesTM generated and analyzed in this study is available in the GitHub repository, https://github.com/phngs/allestm -The datasets used and/or analyzed in this current study are available from the corresponding author upon request
