# Supplementary material for: AllesTM: predicting multiple structural features of transmembrane proteins
Source: BMC Bioinformatics. 2020 Jun 12;21:242. doi: 10.1186/s12859-020-03581-8 (PMC7291640; doi:10.1186/s12859-020-03581-8)
Supplement: Supplementary file 1 — Additional file 1: S1 Fig. Distribution of the observed and predicted z-coordinates on the cross-validation dataset. S2 Fig. Distribution of the observed and predicted z-coordinates on the independent test dataset. S3 Fig. Precision-recall and ROC curves of the predicted topology on the cross-validation dataset. S4 Fig. Precision-recall and ROC curves of the predicted topology on the independent test dataset. S5 Fig. Distribution of the observed and predicted continuous flexibility on the cross-validation dataset. S6 Fig. Distribution of the observed and predicted continuous flexibility on the independent test dataset. S7 Fig. Precision-recall and ROC curve of the predicted two-state flexibility on the cross-validation dataset. S8 Fig. Precision-recall and ROC curve of the predicted two-state flexibility on the independent test dataset. S9 Fig. Distribution of the observed and predicted φ angles on the cross-validation dataset. S10 Fig. Distribution of the observed and predicted φ angles on the independent test dataset. S11 Fig. Distribution of the observed and predicted ψ angles on the cross-validation dataset. S12 Fig. Distribution of the observed and predicted ψ angles on the independent test dataset. S13 Fig. Precision-recall and ROC curves of the predicted secondary structure on the cross-validation dataset. S14 Fig. Precision-recall and ROC curves of the predicted secondary structure on the independent test dataset. S15 Fig. Distribution of the observed and predicted relative solvent accessibility of monomers on the cross-validation dataset. S16 Fig. Distribution of the observed and predicted relative solvent accessibility of monomers on the independent test dataset. S17 Fig. Distribution of the observed and predicted relative solvent accessibility of protein chains in complexes on the cross-validation dataset. S18 Fig. Distribution of the observed and predicted relative solvent accessibility of protein chains in complexes on the independent test dataset. S19 [file 12859_2020_3581_MOESM1_ESM.docx]

**Supplementary tables**

| ***Cross-validation*** | | | | |
| --- | --- | --- | --- | --- |
|  | **r** | **MAE** | **MSE** | **RMSE** |
| **RF** | 0.68 | 10.64 | 185.06 | 13.6 |
| **GBM** | 0.71 | 9.63 | 163.51 | 12.79 |
| **conv** | 0.73 | 6.94 | 155.87 | 12.48 |
| **dconv** | 0.81 | 6.02 | 106.98 | 10.34 |
| **LSTM** | 0.84 | 4.99 | 94.02 | 9.7 |
| **AllesTM** | 0.84 | 5.08 | 91.98 | 9.59 |
|  |  |  |  |  |
| ***Independent test*** | | | | |
|  | **r** | **MAE** | **MSE** | **RMSE** |
| **RF** | 0.79 | 9.1 | 134.69 | 11.61 |
| **GBM** | 0.84 | 7.82 | 106.05 | 10.3 |
| **conv** | 0.87 | 5.18 | 71.3 | 8.44 |
| **dconv** | 0.92 | 4.45 | 47.96 | 6.93 |
| **LSTM** | 0.93 | 3.71 | 40.84 | 6.39 |
| **AllesTM** | 0.93 | 3.72 | 39.96 | 6.32 |

S1 Table - Performance of several algorithms, including the final method AllesTM, for z-coordinate prediction on the cross-validation and independent test datasets.

| ***Cross-validation*** | | | | | | | | | | | | | | | | | | | | | |
| --- | --- | --- | --- | --- | --- | --- | --- | --- | --- | --- | --- | --- | --- | --- | --- | --- | --- | --- | --- | --- | --- |
|  |  | **In** | | | | | **TMS** | | | | | **Out** | | | | | **RER** | | | | |
|  | **ACC** | **P** | **R** | **F1** | **AUC** | **MCC** | **P** | **R** | **F1** | **AUC** | **MCC** | **P** | **R** | **F1** | **AUC** | **MCC** | **P** | **R** | **F1** | **AUC** | **MCC** |
| **RF** | 0.79 | 0.73 | 0.85 | 0.79 | 0.85 | 0.68 | 0.83 | 0.89 | 0.86 | 0.88 | 0.75 | 0.79 | 0.61 | 0.68 | 0.78 | 0.61 | 0.17 | 0 | 0.01 | 0.5 | 0.02 |
| **GBM** | 0.8 | 0.76 | 0.85 | 0.8 | 0.86 | 0.7 | 0.85 | 0.88 | 0.87 | 0.89 | 0.77 | 0.76 | 0.68 | 0.72 | 0.81 | 0.64 | 0.48 | 0.03 | 0.05 | 0.51 | 0.11 |
| **conv** | 0.8 | 0.79 | 0.82 | 0.8 | 0.86 | 0.7 | 0.86 | 0.88 | 0.87 | 0.89 | 0.78 | 0.73 | 0.75 | 0.74 | 0.83 | 0.65 | 0.56 | 0.01 | 0.02 | 0.5 | 0.07 |
| **dconv** | 0.83 | 0.83 | 0.86 | 0.84 | 0.89 | 0.76 | 0.87 | 0.88 | 0.87 | 0.89 | 0.79 | 0.78 | 0.79 | 0.79 | 0.86 | 0.72 | 0.41 | 0.03 | 0.06 | 0.52 | 0.11 |
| **LSTM** | 0.84 | 0.83 | 0.88 | 0.86 | 0.9 | 0.79 | 0.86 | 0.88 | 0.87 | 0.89 | 0.78 | 0.82 | 0.79 | 0.8 | 0.87 | 0.74 | 0.43 | 0.1 | 0.17 | 0.55 | 0.2 |
| **AllesTM** | 0.85 | 0.85 | 0.89 | 0.87 | 0.9 | 0.8 | 0.88 | 0.88 | 0.88 | 0.9 | 0.8 | 0.82 | 0.83 | 0.82 | 0.88 | 0.77 | 0.47 | 0.12 | 0.19 | 0.56 | 0.23 |
| **MEMSAT-SVM** | 0.73 | 0.72 | 0.69 | 0.7 | 0.78 | 0.56 | 0.88 | 0.82 | 0.85 | 0.87 | 0.75 | 0.56 | 0.71 | 0.63 | 0.77 | 0.5 | 0.54 | 0.14 | 0.22 | 0.57 | 0.27 |
| **PolyPhobius** | 0.76 | 0.77 | 0.7 | 0.73 | 0.8 | 0.61 | 0.87 | 0.84 | 0.85 | 0.87 | 0.75 | 0.61 | 0.78 | 0.68 | 0.81 | 0.58 | 0 | 0 | 0 | 0.5 | 0 |
| **SCAMPI** | 0.75 | 0.73 | 0.78 | 0.76 | 0.82 | 0.63 | 0.86 | 0.78 | 0.82 | 0.84 | 0.7 | 0.61 | 0.73 | 0.67 | 0.79 | 0.55 | 0 | 0 | 0 | 0.5 | 0 |
|  |  |  |  |  |  |  |  |  |  |  |  |  |  |  |  |  |  |  |  |  |  |
| ***Independent test*** | | | | | | | | | | | | | | | | | | | | | |
|  |  | **In** | | | | | **TMS** | | | | | **Out** | | | | | **RER** | | | | |
|  | **ACC** | **P** | **R** | **F1** | **AUC** | **MCC** | **P** | **R** | **F1** | **AUC** | **MCC** | **P** | **R** | **F1** | **AUC** | **MCC** | **P** | **R** | **F1** | **AUC** | **MCC** |
| **RF** | 0.83 | 0.83 | 0.87 | 0.85 | 0.89 | 0.77 | 0.84 | 0.91 | 0.87 | 0.89 | 0.77 | 0.81 | 0.67 | 0.73 | 0.82 | 0.68 | 0 | 0 | 0 | 0.5 | 0 |
| **GBM** | 0.86 | 0.87 | 0.89 | 0.88 | 0.91 | 0.82 | 0.87 | 0.9 | 0.88 | 0.9 | 0.79 | 0.82 | 0.78 | 0.8 | 0.87 | 0.75 | 1 | 0.02 | 0.03 | 0.51 | 0.13 |
| **conv** | 0.86 | 0.89 | 0.86 | 0.87 | 0.9 | 0.81 | 0.88 | 0.91 | 0.9 | 0.91 | 0.81 | 0.78 | 0.85 | 0.81 | 0.89 | 0.77 | 0 | 0 | 0 | 0.5 | 0 |
| **dconv** | 0.88 | 0.91 | 0.89 | 0.9 | 0.92 | 0.84 | 0.89 | 0.92 | 0.9 | 0.91 | 0.83 | 0.82 | 0.86 | 0.84 | 0.91 | 0.8 | 0.97 | 0.16 | 0.28 | 0.58 | 0.39 |
| **LSTM** | 0.89 | 0.9 | 0.91 | 0.91 | 0.93 | 0.86 | 0.89 | 0.92 | 0.91 | 0.92 | 0.83 | 0.86 | 0.83 | 0.84 | 0.9 | 0.8 | 0.87 | 0.3 | 0.45 | 0.65 | 0.51 |
| **AllesTM** | 0.9 | 0.91 | 0.92 | 0.91 | 0.94 | 0.87 | 0.9 | 0.92 | 0.91 | 0.92 | 0.83 | 0.86 | 0.86 | 0.86 | 0.91 | 0.83 | 0.88 | 0.4 | 0.55 | 0.7 | 0.59 |
| **MEMSAT-SVM** | 0.74 | 0.7 | 0.76 | 0.73 | 0.8 | 0.58 | 0.91 | 0.85 | 0.88 | 0.89 | 0.79 | 0.47 | 0.52 | 0.49 | 0.69 | 0.36 | 0.81 | 0.16 | 0.27 | 0.58 | 0.36 |
| **PolyPhobius** | 0.75 | 0.77 | 0.68 | 0.72 | 0.78 | 0.59 | 0.87 | 0.86 | 0.86 | 0.88 | 0.76 | 0.52 | 0.7 | 0.6 | 0.77 | 0.49 | 0 | 0 | 0 | 0.5 | 0 |
| **SCAMPI** | 0.77 | 0.78 | 0.82 | 0.8 | 0.85 | 0.69 | 0.86 | 0.8 | 0.83 | 0.85 | 0.71 | 0.6 | 0.69 | 0.64 | 0.79 | 0.55 | 0 | 0 | 0 | 0.5 | 0 |

S2 Table - Protein topology prediction performance.

| ***Cross-validation*** | | | | |
| --- | --- | --- | --- | --- |
|  | **r** | **MAE** | **MSE** | **RMSE** |
| **RF** | 0.42 | 0.69 | 0.82 | 0.91 |
| **GBM** | 0.44 | 0.67 | 0.81 | 0.9 |
| **conv** | 0.45 | 0.66 | 0.82 | 0.91 |
| **dconv** | 0.46 | 0.66 | 0.8 | 0.9 |
| **LSTM** | 0.45 | 0.66 | 0.81 | 0.9 |
| **AllesTM** | 0.48 | 0.66 | 0.77 | 0.88 |
| **PROFbval** | 0.43 | 0.77 | 1 | 1 |
| **PredyFlexy** | 0.14 | 0.81 | 1.08 | 1.04 |
|  |  |  |  |  |
| ***Independent test*** | | | | |
|  | **r** | **MAE** | **MSE** | **RMSE** |
| **RF** | 0.44 | 0.67 | 0.81 | 0.9 |
| **GBM** | 0.47 | 0.65 | 0.78 | 0.89 |
| **conv** | 0.5 | 0.63 | 0.78 | 0.88 |
| **dconv** | 0.5 | 0.63 | 0.77 | 0.88 |
| **LSTM** | 0.5 | 0.63 | 0.78 | 0.88 |
| **AllesTM** | 0.51 | 0.63 | 0.75 | 0.86 |
| **PROFbval** | 0.4 | 0.78 | 1.07 | 1.04 |
| **PredyFlexy** | 0.13 | 0.8 | 1.09 | 1.04 |

**S3 Table - Continuous flexibility prediction performance.**

| ***Cross-validation*** | | | | | | |
| --- | --- | --- | --- | --- | --- | --- |
|  | **ACC** | **P** | **R** | **F1** | **AUC** | **MCC** |
| **RF** | 0.68 | 0.62 | 0.46 | 0.53 | 0.64 | 0.3 |
| **GBM** | 0.67 | 0.6 | 0.48 | 0.53 | 0.64 | 0.29 |
| **conv** | 0.68 | 0.64 | 0.45 | 0.53 | 0.64 | 0.31 |
| **dconv** | 0.68 | 0.62 | 0.5 | 0.56 | 0.65 | 0.31 |
| **LSTM** | 0.68 | 0.62 | 0.49 | 0.55 | 0.65 | 0.31 |
| **AllesTM** | 0.69 | 0.63 | 0.49 | 0.55 | 0.65 | 0.32 |
| **PROFbval** | 0.65 | 0.68 | 0.21 | 0.32 | 0.57 | 0.22 |
|  |  |  |  |  |  |  |
| ***Independent test*** | | | | | | |
|  | **ACC** | **P** | **R** | **F1** | **AUC** | **MCC** |
| **RF** | 0.68 | 0.62 | 0.46 | 0.53 | 0.64 | 0.31 |
| **GBM** | 0.69 | 0.63 | 0.47 | 0.54 | 0.65 | 0.32 |
| **conv** | 0.7 | 0.65 | 0.46 | 0.54 | 0.65 | 0.34 |
| **dconv** | 0.7 | 0.63 | 0.52 | 0.57 | 0.66 | 0.34 |
| **LSTM** | 0.7 | 0.64 | 0.48 | 0.55 | 0.66 | 0.33 |
| **AllesTM** | 0.7 | 0.64 | 0.49 | 0.56 | 0.66 | 0.34 |
| **PROFbval** | 0.65 | 0.66 | 0.19 | 0.29 | 0.56 | 0.2 |

S4 Table - Two-state flexibility prediction performance.

| ***Cross-validation*** | | | | |
| --- | --- | --- | --- | --- |
|  | **r** | **MAE** | **MSE** | **RMSE** |
| **RF** | 0.53 | 20.72 | 1259.5 | 35.49 |
| **GBM** | 0.54 | 20.4 | 1241.78 | 35.24 |
| **conv** | 0.53 | 19.44 | 1282.17 | 35.81 |
| **dconv** | 0.52 | 19.6 | 1299.52 | 36.05 |
| **LSTM** | 0.52 | 19.31 | 1319.27 | 36.32 |
| **AllesTM** | 0.57 | 19.13 | 1188.15 | 34.47 |
| **SPINE X** | 0.49 | 20.64 | 1474.41 | 38.4 |
| **ANGLOR** | 0.43 | 21.25 | 1492.83 | 38.64 |
| **SPOT-1D** | 0.6 | 17 | 1204.58 | 34.71 |
|  |  |  |  |  |
| ***Independent test*** | | | | |
|  | **r** | **MAE** | **MSE** | **RMSE** |
| **RF** | 0.55 | 18.91 | 1039.18 | 32.24 |
| **GBM** | 0.57 | 18.5 | 1012.28 | 31.82 |
| **conv** | 0.59 | 16.93 | 970.28 | 31.15 |
| **dconv** | 0.56 | 17.43 | 1026.22 | 32.03 |
| **LSTM** | 0.57 | 17.03 | 1000.33 | 31.63 |
| **AllesTM** | 0.59 | 17.34 | 962.86 | 31.03 |
| **SPINE X** | 0.45 | 20.68 | 1409.31 | 37.54 |
| **ANGLOR** | 0.45 | 19.57 | 1246.15 | 35.3 |
| **SPOT**-1D | 0.62 | 15.85 | 1004.36 | 31.69 |

**S5 Table - ϕ angles performance.**

| ***Cross-validation*** | | | | |
| --- | --- | --- | --- | --- |
|  | **r** | **MAE** | **MSE** | **RMSE** |
| **RF** | 0.61 | 42.91 | 3710.94 | 60.92 |
| **GBM** | 0.64 | 39.37 | 3365.24 | 58.01 |
| **conv** | 0.65 | 33.21 | 3465.95 | 58.87 |
| **dconv** | 0.62 | 34.24 | 3711.37 | 60.92 |
| **LSTM** | 0.62 | 33.25 | 3709.67 | 60.91 |
| **AllesTM** | 0.69 | 33.57 | 3021.61 | 54.97 |
| **SPINE X** | 0.52 | 39.76 | 5596.51 | 74.81 |
| **ANGLOR** | 0.61 | 40.47 | 3711.86 | 60.93 |
| **SPOT-1D** | 0.73 | 25.71 | 2949.86 | 54.31 |
|  |  |  |  |  |
| ***Independent test*** | | | | |
|  | **r** | **MAE** | **MSE** | **RMSE** |
| **RF** | 0.62 | 39.5 | 3228.61 | 56.82 |
| **GBM** | 0.66 | 35.93 | 2893.74 | 53.79 |
| **conv** | 0.7 | 28.75 | 2648.83 | 51.47 |
| **dconv** | 0.68 | 29.47 | 2740.61 | 52.35 |
| **LSTM** | 0.68 | 28.81 | 2762.24 | 52.56 |
| **AllesTM** | 0.7 | 30.41 | 2573.11 | 50.73 |
| **SPINE X** | 0.46 | 41.77 | 5991.92 | 77.41 |
| **ANGLOR** | 0.61 | 38.33 | 3350.11 | 57.88 |
| **SPOT-1D** | 0.74 | 23.51 | 2511.18 | 50.11 |

S6 Table - ψ angles performance.

| ***Cross-validation*** | | | | | | | | | | | | | | | | | | | | | |  |  |  |
| --- | --- | --- | --- | --- | --- | --- | --- | --- | --- | --- | --- | --- | --- | --- | --- | --- | --- | --- | --- | --- | --- | --- | --- | --- |
|  |  | **Helix** | | | | | **Strand** | | | | | | | **Coil** | | | | | | | | |  |  |
|  | **ACC** | **P** | **R** | **F1** | **AUC** | **MCC** | **P** | | **R** | | **F1** | **AUC** | **MCC** | **P** | **R** | | **F1** | | **AUC** | | **MCC** | | |  |
| **RF** | 0.8 | 0.83 | 0.9 | 0.87 | 0.8 | 0.62 | 0.93 | 0.22 | | 0.36 | | 0.61 | 0.44 | 0.71 | 0.72 | 0.71 | | 0.79 | | 0.59 | | | | |
| **GBM** | 0.82 | 0.87 | 0.9 | 0.88 | 0.84 | 0.68 | 0.84 | 0.42 | | 0.56 | | 0.71 | 0.58 | 0.71 | 0.76 | 0.73 | | 0.81 | | 0.62 | | | | |
| **conv** | 0.83 | 0.9 | 0.89 | 0.9 | 0.86 | 0.73 | 0.66 | 0.61 | | 0.64 | | 0.79 | 0.61 | 0.73 | 0.76 | 0.74 | | 0.82 | | 0.63 | | | | |
| **dconv** | 0.83 | 0.89 | 0.9 | 0.9 | 0.86 | 0.72 | 0.7 | 0.62 | | 0.66 | | 0.8 | 0.63 | 0.74 | 0.74 | 0.74 | | 0.81 | | 0.63 | | | | |
| **LSTM** | 0.83 | 0.89 | 0.89 | 0.89 | 0.85 | 0.71 | 0.71 | 0.53 | | 0.61 | | 0.76 | 0.59 | 0.72 | 0.76 | 0.74 | | 0.82 | | 0.63 | | | | |
| **AllesTM** | 0.84 | 0.89 | 0.91 | 0.9 | 0.87 | 0.74 | 0.73 | 0.64 | | 0.68 | | 0.81 | 0.66 | 0.75 | 0.75 | 0.75 | | 0.82 | | 0.64 | | | | |
| **SPINE X** | 0.79 | 0.87 | 0.87 | 0.87 | 0.82 | 0.65 | 0.51 | 0.56 | | 0.53 | | 0.76 | 0.49 | 0.69 | 0.68 | 0.69 | | 0.78 | | 0.55 | | | | |
| **PROFphd** | 0.77 | 0.88 | 0.82 | 0.85 | 0.82 | 0.63 | 0.44 | 0.63 | | 0.52 | | 0.78 | 0.48 | 0.69 | 0.71 | 0.7 | | 0.79 | | 0.57 | | | | |
| **PSIPRED** | 0.85 | 0.9 | 0.92 | 0.91 | 0.88 | 0.76 | 0.78 | 0.66 | | 0.72 | | 0.82 | 0.7 | 0.77 | 0.77 | 0.77 | | 0.84 | | 0.67 | | | | |
| **SPOT-1D** | 0.88 | 0.9 | 0.95 | 0.93 | 0.89 | 0.8 | 0.84 | 0.77 | | 0.8 | | 0.88 | 0.79 | 0.84 | 0.76 | 0.8 | | 0.85 | | 0.72 | | | | |

|  | |  |  |  |  |  |  |  | |  | |  | |  | |  | | |  | | |  |  | |  | |  | | |  |  |  |
| --- | --- | --- | --- | --- | --- | --- | --- | --- | --- | --- | --- | --- | --- | --- | --- | --- | --- | --- | --- | --- | --- | --- | --- | --- | --- | --- | --- | --- | --- | --- | --- | --- |
| ***Independent test*** | | | | | | | | | | | | | | | | | | | | | | | | | | | | |  |  |  |  |
|  |  | | | **Helix** | | | | | | | **Strand** | | | | | | | | | **Coil** | | | | | | | | | | | | |
|  | **ACC** | | | **P** | **R** | **F1** | **AUC** | | **MCC** | | **P** | | **R** | | **F1** | | **AUC** | **MCC** | | | **P** | | | **R** | | **F1** | | **AUC** | | | **MCC** |  |
| **RF** | 0.82 | | | 0.86 | 0.9 | 0.88 | 0.8 | | 0.62 | | 0.97 | | 0.23 | | 0.37 | | 0.62 | 0.46 | | | 0.69 | | | 0.72 | | 0.7 | | 0.8 | | | 0.59 |  |
| **GBM** | 0.84 | | | 0.9 | 0.9 | 0.9 | 0.84 | | 0.68 | | 0.91 | | 0.46 | | 0.61 | | 0.73 | 0.64 | | | 0.7 | | | 0.77 | | 0.73 | | 0.82 | | | 0.63 |  |
| **conv** | 0.85 | | | 0.92 | 0.9 | 0.91 | 0.87 | | 0.72 | | 0.77 | | 0.62 | | 0.68 | | 0.8 | 0.67 | | | 0.71 | | | 0.79 | | 0.75 | | 0.84 | | | 0.65 |  |
| **dconv** | 0.85 | | | 0.9 | 0.91 | 0.91 | 0.85 | | 0.71 | | 0.77 | | 0.61 | | 0.68 | | 0.8 | 0.67 | | | 0.73 | | | 0.74 | | 0.74 | | 0.82 | | | 0.64 |  |
| **LSTM** | 0.85 | | | 0.92 | 0.9 | 0.91 | 0.86 | | 0.72 | | 0.81 | | 0.58 | | 0.67 | | 0.79 | 0.67 | | | 0.72 | | | 0.79 | | 0.75 | | 0.84 | | | 0.65 |  |
| **AllesTM** | 0.86 | | | 0.91 | 0.91 | 0.91 | 0.86 | | 0.72 | | 0.77 | | 0.62 | | 0.69 | | 0.8 | 0.68 | | | 0.73 | | | 0.76 | | 0.74 | | 0.83 | | | 0.65 |  |
| **SPINE X** | 0.77 | | | 0.88 | 0.83 | 0.85 | 0.8 | | 0.57 | | 0.32 | | 0.47 | | 0.38 | | 0.71 | 0.34 | | | 0.65 | | | 0.69 | | 0.67 | | 0.78 | | | 0.55 |  |
| **PROFphd** | 0.78 | | | 0.91 | 0.82 | 0.86 | 0.82 | | 0.61 | | 0.35 | | 0.57 | | 0.43 | | 0.76 | 0.41 | | | 0.65 | | | 0.73 | | 0.69 | | 0.8 | | | 0.57 |  |
| **PSIPRED** | 0.87 | | | 0.92 | 0.92 | 0.92 | 0.88 | | 0.76 | | 0.82 | | 0.6 | | 0.69 | | 0.8 | 0.69 | | | 0.75 | | | 0.79 | | 0.77 | | 0.85 | | | 0.68 |  |
| **SPOT-1D** | 0.89 | | | 0.92 | 0.94 | 0.93 | 0.89 | | 0.78 | | 0.86 | | 0.76 | | 0.81 | | 0.88 | 0.8 | | | 0.8 | | | 0.78 | | 0.79 | | 0.86 | | | 0.72 |  |

S7 Table - Secondary structure prediction performance.

| ***Cross-validation*** | | | | | | | | | | | | | | | | |
| --- | --- | --- | --- | --- | --- | --- | --- | --- | --- | --- | --- | --- | --- | --- | --- | --- |
|  |  | **H** | | | | | **E** | | | | | **C** | | | | |
|  | **ACC** | **P** | **R** | **F1** | **AUC** | **MCC** | **P** | **R** | **F1** | **AUC** | **MCC** | **P** | **R** | **F1** | **AUC** | **MCC** |
| **RF** | 0.71 | 0.68 | 0.79 | 0.73 | 0.76 | 0.52 | 0.94 | 0.23 | 0.37 | 0.61 | 0.44 | 0.72 | 0.77 | 0.74 | 0.76 | 0.52 |
| **GBM** | 0.74 | 0.76 | 0.77 | 0.76 | 0.8 | 0.59 | 0.84 | 0.44 | 0.58 | 0.71 | 0.57 | 0.72 | 0.8 | 0.76 | 0.77 | 0.54 |
| **conv** | 0.76 | 0.81 | 0.77 | 0.79 | 0.82 | 0.65 | 0.67 | 0.62 | 0.65 | 0.79 | 0.6 | 0.74 | 0.79 | 0.77 | 0.78 | 0.56 |
| **dconv** | 0.77 | 0.8 | 0.79 | 0.8 | 0.82 | 0.65 | 0.7 | 0.63 | 0.66 | 0.79 | 0.62 | 0.75 | 0.78 | 0.77 | 0.78 | 0.56 |
| **LSTM** | 0.75 | 0.79 | 0.77 | 0.78 | 0.81 | 0.62 | 0.72 | 0.54 | 0.62 | 0.76 | 0.58 | 0.73 | 0.8 | 0.77 | 0.78 | 0.55 |
| **AllesTM** | 0.78 | 0.81 | 0.8 | 0.81 | 0.83 | 0.67 | 0.73 | 0.65 | 0.69 | 0.81 | 0.65 | 0.76 | 0.79 | 0.78 | 0.79 | 0.58 |
| **SPINE X** | 0.73 | 0.76 | 0.79 | 0.77 | 0.8 | 0.61 | 0.6 | 0.56 | 0.58 | 0.75 | 0.52 | 0.74 | 0.73 | 0.73 | 0.75 | 0.51 |
| **PROFphd** | 0.73 | 0.78 | 0.74 | 0.76 | 0.8 | 0.6 | 0.61 | 0.63 | 0.62 | 0.78 | 0.56 | 0.72 | 0.76 | 0.74 | 0.76 | 0.51 |
| **PSIPRED** | 0.8 | 0.83 | 0.84 | 0.83 | 0.86 | 0.71 | 0.78 | 0.67 | 0.72 | 0.82 | 0.69 | 0.79 | 0.81 | 0.8 | 0.81 | 0.62 |
| **SPOT-1D** | 0.84 | 0.83 | 0.9 | 0.86 | 0.88 | 0.76 | 0.84 | 0.78 | 0.81 | 0.88 | 0.78 | 0.85 | 0.8 | 0.82 | 0.84 | 0.68 |
|  |  |  |  |  |  |  |  |  |  |  |  |  |  |  |  |  |
| ***Independent test*** | | | | | | | | | | | | | | | | |
|  |  | **H** | | | | | **E** | | | | | **C** | | | | |
|  | **ACC** | **P** | **R** | **F1** | **AUC** | **MCC** | **P** | **R** | **F1** | **AUC** | **MCC** | **P** | **R** | **F1** | **AUC** | **MCC** |
| **RF** | 0.73 | 0.74 | 0.78 | 0.76 | 0.76 | 0.52 | 0.97 | 0.24 | 0.39 | 0.62 | 0.47 | 0.7 | 0.78 | 0.74 | 0.77 | 0.53 |
| **GBM** | 0.77 | 0.82 | 0.77 | 0.79 | 0.8 | 0.61 | 0.92 | 0.48 | 0.63 | 0.74 | 0.64 | 0.71 | 0.83 | 0.76 | 0.79 | 0.57 |
| **conv** | 0.78 | 0.86 | 0.77 | 0.81 | 0.82 | 0.65 | 0.78 | 0.64 | 0.7 | 0.81 | 0.68 | 0.72 | 0.84 | 0.77 | 0.8 | 0.59 |
| **dconv** | 0.78 | 0.82 | 0.8 | 0.81 | 0.82 | 0.64 | 0.77 | 0.63 | 0.69 | 0.81 | 0.67 | 0.73 | 0.79 | 0.76 | 0.79 | 0.58 |
| **LSTM** | 0.78 | 0.85 | 0.78 | 0.81 | 0.82 | 0.65 | 0.81 | 0.6 | 0.69 | 0.79 | 0.67 | 0.72 | 0.83 | 0.77 | 0.8 | 0.59 |
| **AllesTM** | 0.79 | 0.84 | 0.8 | 0.82 | 0.83 | 0.66 | 0.77 | 0.64 | 0.7 | 0.81 | 0.68 | 0.74 | 0.81 | 0.77 | 0.8 | 0.59 |
| **SPINE X** | 0.72 | 0.77 | 0.76 | 0.77 | 0.78 | 0.55 | 0.5 | 0.48 | 0.49 | 0.71 | 0.44 | 0.71 | 0.72 | 0.72 | 0.75 | 0.5 |
| **PROFphd** | 0.73 | 0.82 | 0.72 | 0.77 | 0.78 | 0.57 | 0.57 | 0.57 | 0.57 | 0.76 | 0.52 | 0.68 | 0.77 | 0.72 | 0.75 | 0.5 |
| **PSIPRED** | 0.81 | 0.87 | 0.84 | 0.85 | 0.86 | 0.72 | 0.82 | 0.62 | 0.7 | 0.8 | 0.69 | 0.76 | 0.83 | 0.79 | 0.82 | 0.63 |
| **SPOT-1D** | 0.84 | 0.86 | 0.87 | 0.86 | 0.87 | 0.74 | 0.88 | 0.78 | 0.83 | 0.88 | 0.81 | 0.81 | 0.82 | 0.81 | 0.84 | 0.67 |

S8 Table - Secondary structure prediction performance excluding residues situated in transmembrane segments.

| ***Cross-validation*** | | | | |
| --- | --- | --- | --- | --- |
|  | **r** | **MAE** | **MSE** | **RMSE** |
| **RF** | 0.58 | 0.18 | 0.05 | 0.22 |
| **GBM** | 0.62 | 0.17 | 0.04 | 0.21 |
| **conv** | 0.59 | 0.17 | 0.05 | 0.22 |
| **dconv** | 0.61 | 0.16 | 0.05 | 0.22 |
| **LSTM** | 0.61 | 0.16 | 0.05 | 0.22 |
| **AllesTM** | 0.65 | 0.16 | 0.04 | 0.2 |
| **SPINE X** | 0.51 | 0.19 | 0.07 | 0.26 |
| **SPOT-1D** | 0.73 | 0.14 | 0.03 | 0.18 |
|  |  |  |  |  |
| ***Independent test*** | | | | |
|  | **r** | **MAE** | **MSE** | **RMSE** |
| **RF** | 0.57 | 0.18 | 0.05 | 0.22 |
| **GBM** | 0.63 | 0.17 | 0.04 | 0.21 |
| **conv** | 0.61 | 0.16 | 0.05 | 0.22 |
| **dconv** | 0.65 | 0.16 | 0.04 | 0.21 |
| **LSTM** | 0.64 | 0.16 | 0.04 | 0.21 |
| **AllesTM** | 0.67 | 0.15 | 0.04 | 0.2 |
| **SPINE X** | 0.46 | 0.2 | 0.07 | 0.26 |
| **SPOT-1D** | 0.71 | 0.14 | 0.03 | 0.19 |

S9 Table- Monomer solvent accessibility performance.

| ***Cross-validation*** | | | | |
| --- | --- | --- | --- | --- |
|  | **r** | **MAE** | **MSE** | **RMSE** |
| **RF** | 0.6 | 0.16 | 0.04 | 0.2 |
| **GBM** | 0.63 | 0.15 | 0.04 | 0.19 |
| **conv** | 0.63 | 0.15 | 0.04 | 0.2 |
| **dconv** | 0.63 | 0.15 | 0.04 | 0.2 |
| **LSTM** | 0.63 | 0.15 | 0.04 | 0.2 |
| **AllesTM** | 0.67 | 0.14 | 0.04 | 0.19 |
| **SPINE X** | 0.54 | 0.17 | 0.05 | 0.22 |
| **SPOT-1D** | 0.67 | 0.14 | 0.04 | 0.19 |
|  |  |  |  |  |
| ***Independent test*** |  |  |  |  |
|  | **r** | **MAE** | **MSE** | **RMSE** |
| **RF** | 0.59 | 0.16 | 0.04 | 0.2 |
| **GBM** | 0.63 | 0.15 | 0.04 | 0.19 |
| **conv** | 0.63 | 0.14 | 0.04 | 0.19 |
| **dconv** | 0.64 | 0.14 | 0.04 | 0.19 |
| **LSTM** | 0.65 | 0.14 | 0.04 | 0.19 |
| **AllesTM** | 0.66 | 0.14 | 0.03 | 0.19 |
| **SPINE X** | 0.5 | 0.17 | 0.05 | 0.22 |
| **SPOT-1D** | 0.61 | 0.16 | 0.04 | 0.21 |

**S10 Table - Complex solvent accessibility performance.**

| ***Cross-validation*** | | | | |
| --- | --- | --- | --- | --- |
|  | **r** | **MAE** | **MSE** | **RMSE** |
| **RF** | 0.27 | 0.08 | 0.02 | 0.13 |
| **GBM** | 0.3 | 0.08 | 0.02 | 0.13 |
| **conv** | 0.09 | 0.05 | 0.02 | 0.15 |
| **dconv** | 0.11 | 0.06 | 0.02 | 0.15 |
| **LSTM** | 0.12 | 0.05 | 0.02 | 0.15 |
| **AllesTM** | 0.29 | 0.08 | 0.02 | 0.13 |
|  |  |  |  |  |
| ***Independent test*** |  |  |  |  |
|  | **r** | **MAE** | **MSE** | **RMSE** |
| **RF** | 0.32 | 0.09 | 0.02 | 0.15 |
| **GBM** | 0.33 | 0.09 | 0.02 | 0.15 |
| **conv** | 0.16 | 0.07 | 0.03 | 0.17 |
| **dconv** | 0.33 | 0.07 | 0.03 | 0.17 |
| **LSTM** | 0.22 | 0.07 | 0.03 | 0.17 |
| **AllesTM** | 0.39 | 0.08 | 0.02 | 0.15 |

S11 Table - Change of solvent accessibility performance.

| **Fold** | **Training** | **Validation** | **Testing** | **Total** |
| --- | --- | --- | --- | --- |
| 1 | 99 | 15 | 39 | 153 |
| 2 | 101 | 14 | 36 | 151 |
| 3 | 101 | 15 | 37 | 153 |
| 4 | 108 | 14 | 33 | 155 |
| 5 | 110 | 15 | 33 | 158 |

S12 Table - Number of sequences in the training, validation, and test datasets across the five folds. Because of the two-step redundancy reduction procedure, the number of proteins in the validation and training parts are dependent on the test data in the particular fold.

| **Parameter** | **Notation** | **Conv layers** | **Dconv blocks** | **LSTM layers** |
| --- | --- | --- | --- | --- |
| Window size | *w* | 5, 9, 13, 17, 21, 25, 29 | 5, 9 | 1 |
| Embedding size | *n_embedding_* | 5, 10, 15, 20 | | |
| Number of hidden neurons | *n_hidden_neurons_* | 64, 128, 256, 512 | 64, 128, 256 | 64, 128, 256 |
| Number of hidden layers/blocks | *n_hidden_layers_* | 1, 2, 3, 4, 5 | 1, 2 | 1, 2, 3, 4, 5 |
| Number of dconv layers per block | *n_dconv_layers_* | - | 2, 4, 6 | - |
| Residual connections | *-* | None, add, concatenate | | |
| Number of dense layers | *n_dense_layers_* | 0, 1, 2 | | |
| Number of dense neurons | *n_dense_neurons_* | 128, 256, 512, 1024 | | |
| Batch size | *-* | 4, 8, 16, 32 | | |
| Learning rate | *r* | 0.1, 0.01, 0.001 | | |

S13 Table - Parameter values used for the different layer types during random parameter search.

| **Parameter** | **Notation** | **Values** |
| --- | --- | --- |
| Window size | *w* | 5, 9, 13, 17, 21, 25, 29 |
| Fraction of features | *n_features_* | $\sqrt{\#Features}$, 0.1, 0.2, …, 0.9, 1.0 |
| Minimum samples pear leaf | *n_min_samples_* | 1, 3, 5, 9 |

S14 Table - Parameter values used during grid search of random forest models.

| **Parameter** | **Notation** | **Values** |
| --- | --- | --- |
| Window size | *w* | 5, 9, 11, 13, 17, 21, 25, 39 |
| Learning rate | *r* | 0.3, 0.2, 0.1, 0.05, 0.01 |
| Fraction of features | *n_features_* | 0.5, 0.6, 0.7,0.8,0.9,1.0 |
| Fraction of samples | *n_samples_* | 0.5, 0.6, 0.7,0.8,0.9,1.0 |
| Maximum depth | *n_depth_* | 3, 5, 7, 9, 11, 13 |
| Minimum child weight | *n_min_child_weight_* | 1, 3, 5, 7 |
| Gamma | γ | 0.0, 0.1, 0.2, …, 0.8, 0.1, 1.0 |

S15 Table - Parameter values used during random search of GBM models.

**Supplementary figures**


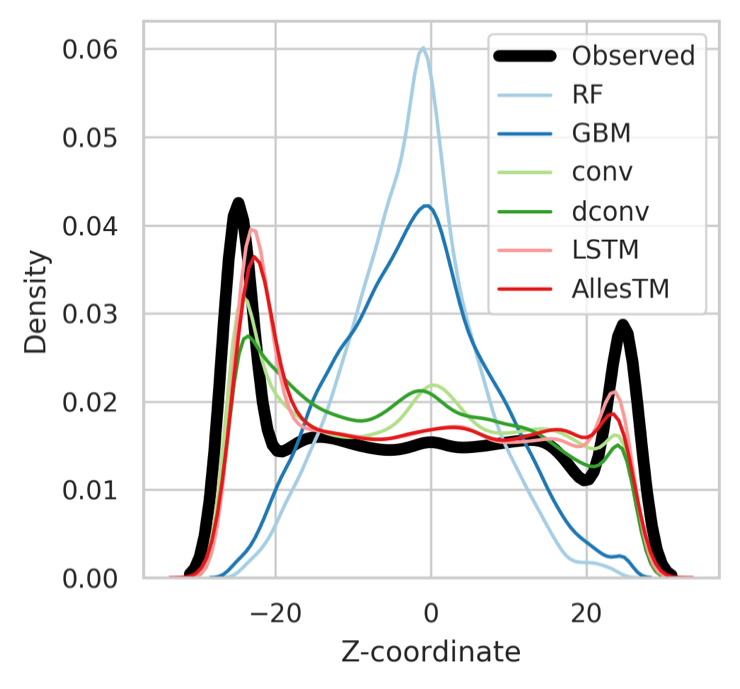


S1 Fig - Distribution of the observed and predicted z-coordinates on the cross-validation dataset.


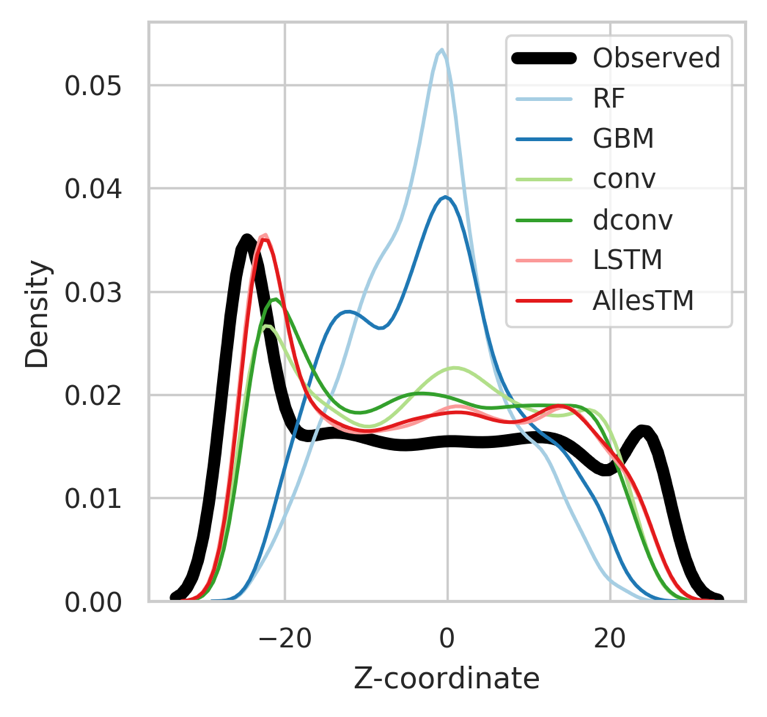


S2 Fig - Distribution of the observed and predicted z-coordinates on the independent test dataset.


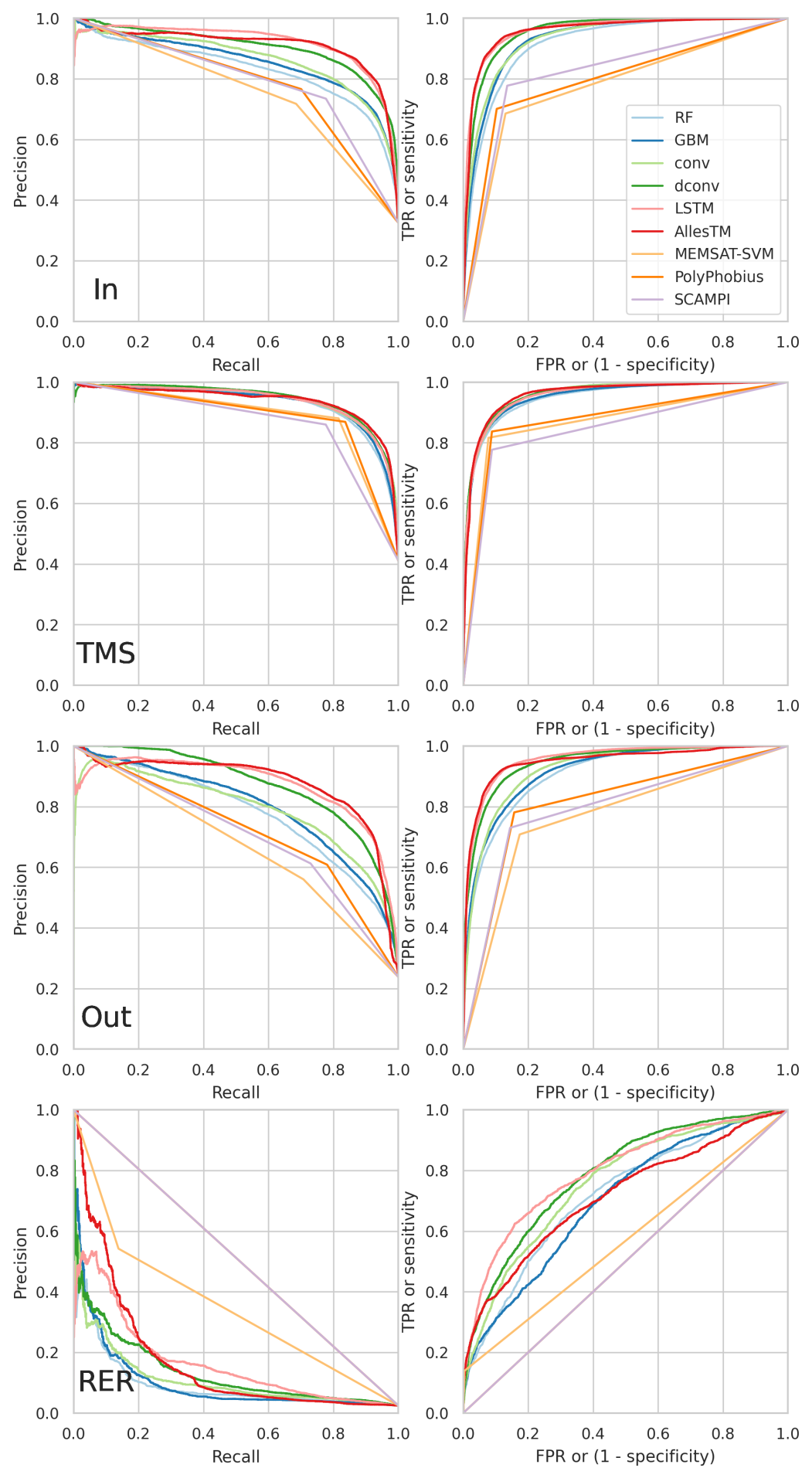


S3 Fig - Precision-recall and ROC curves of the predicted topology on the cross-validation dataset.


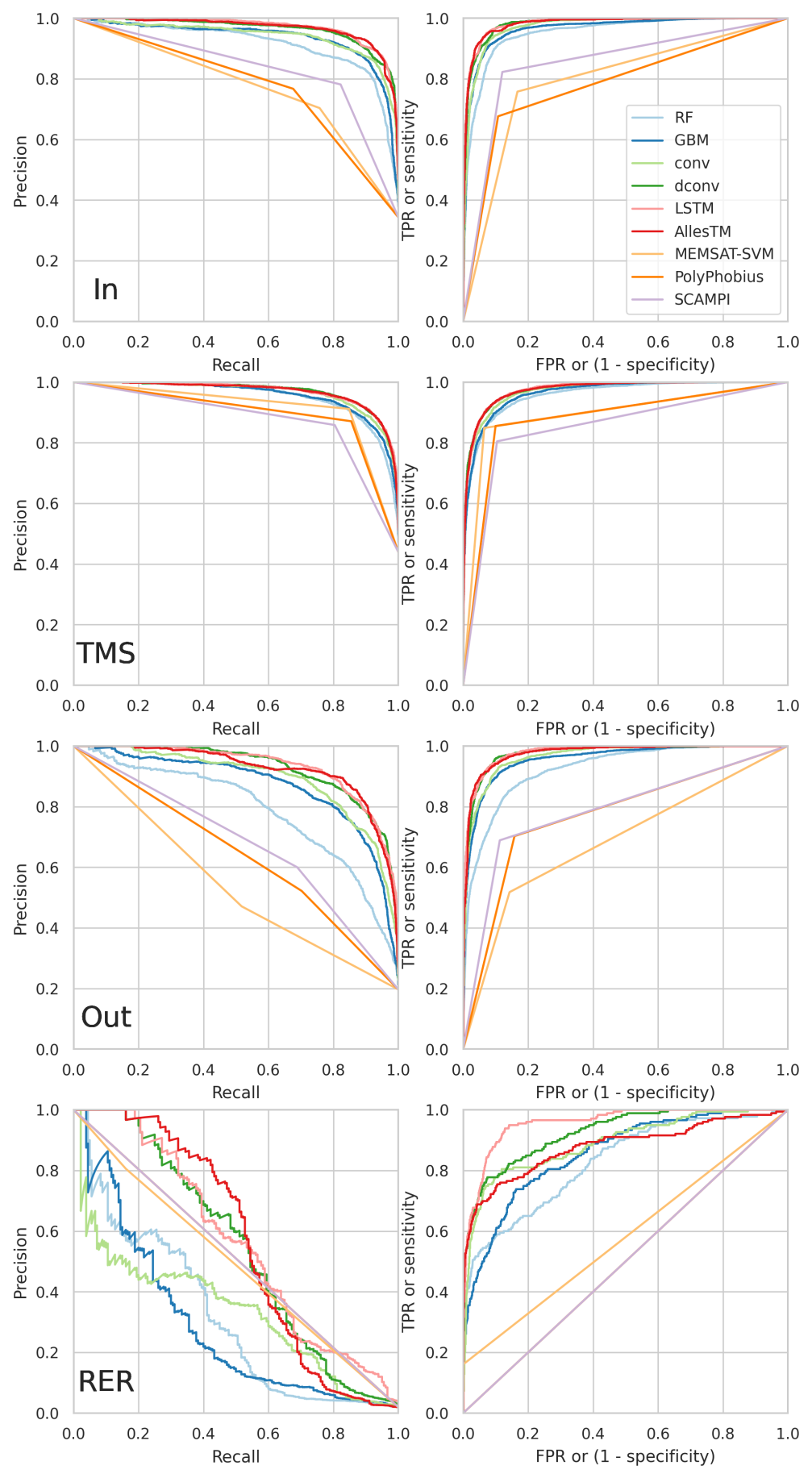


S4 Fig - Precision-recall and ROC curves of the predicted topology on the independent test dataset.


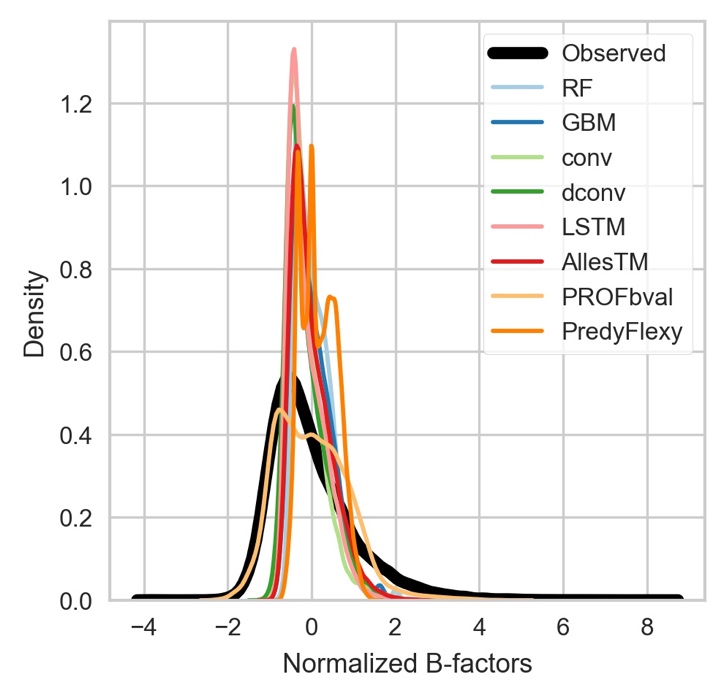


S5 Fig - Distribution of the observed and predicted continuous flexibility on the cross-validation dataset.


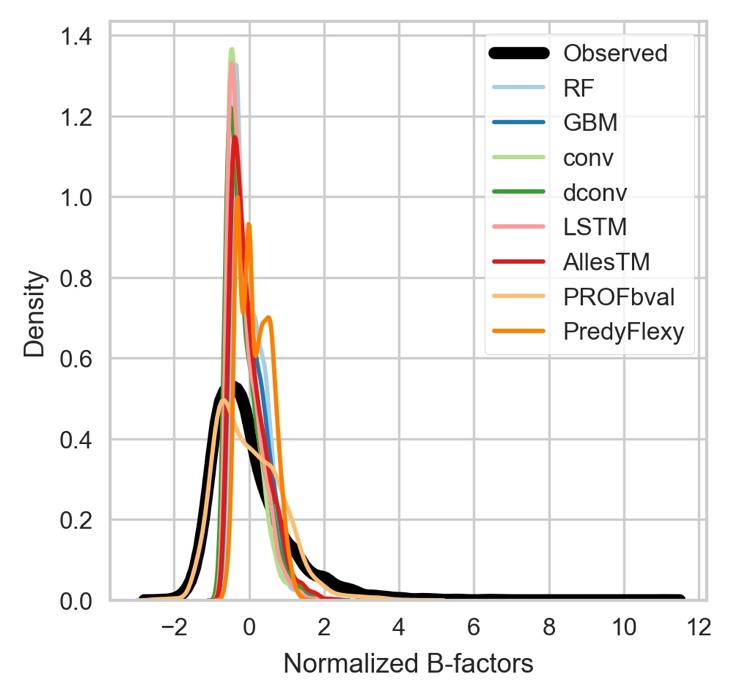


S6 Fig - Distribution of the observed and predicted continuous flexibility on the independent test dataset.


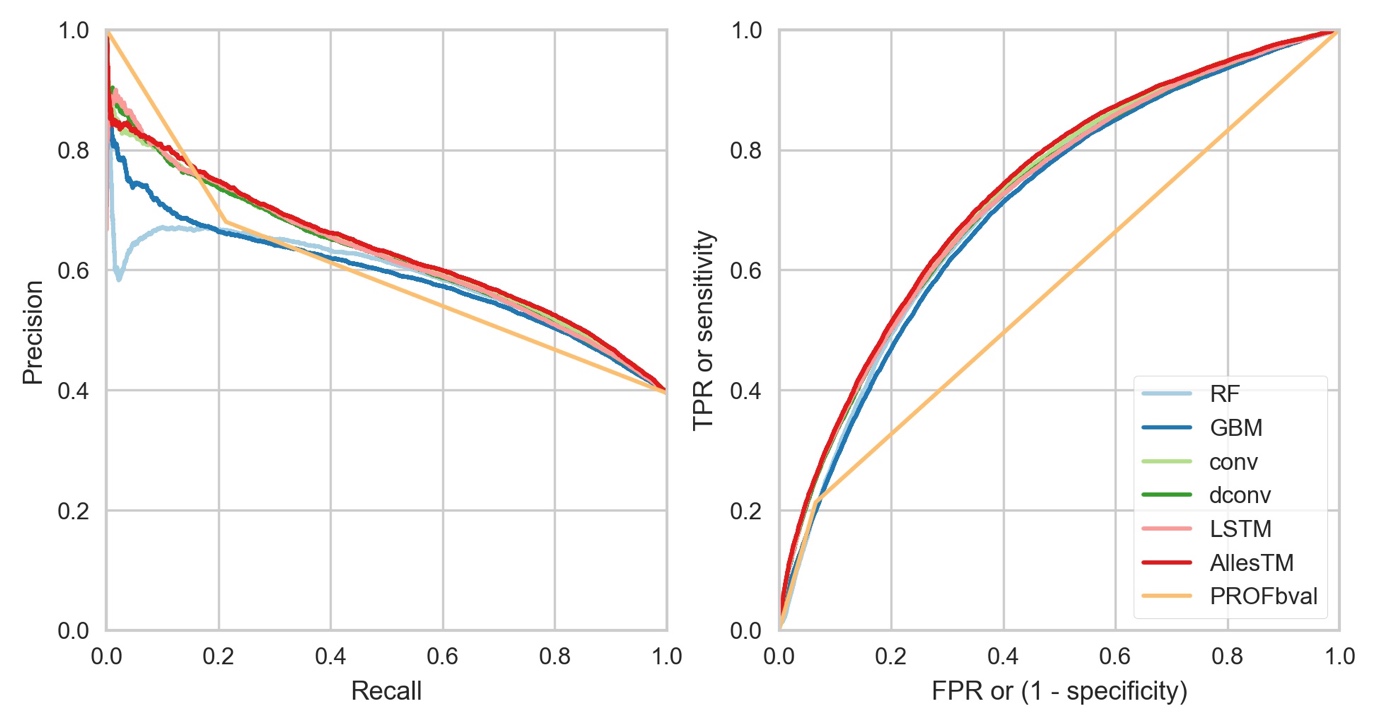


S7 Fig - Precision-recall and ROC curve of the predicted two-state flexibility on the cross-validation dataset.


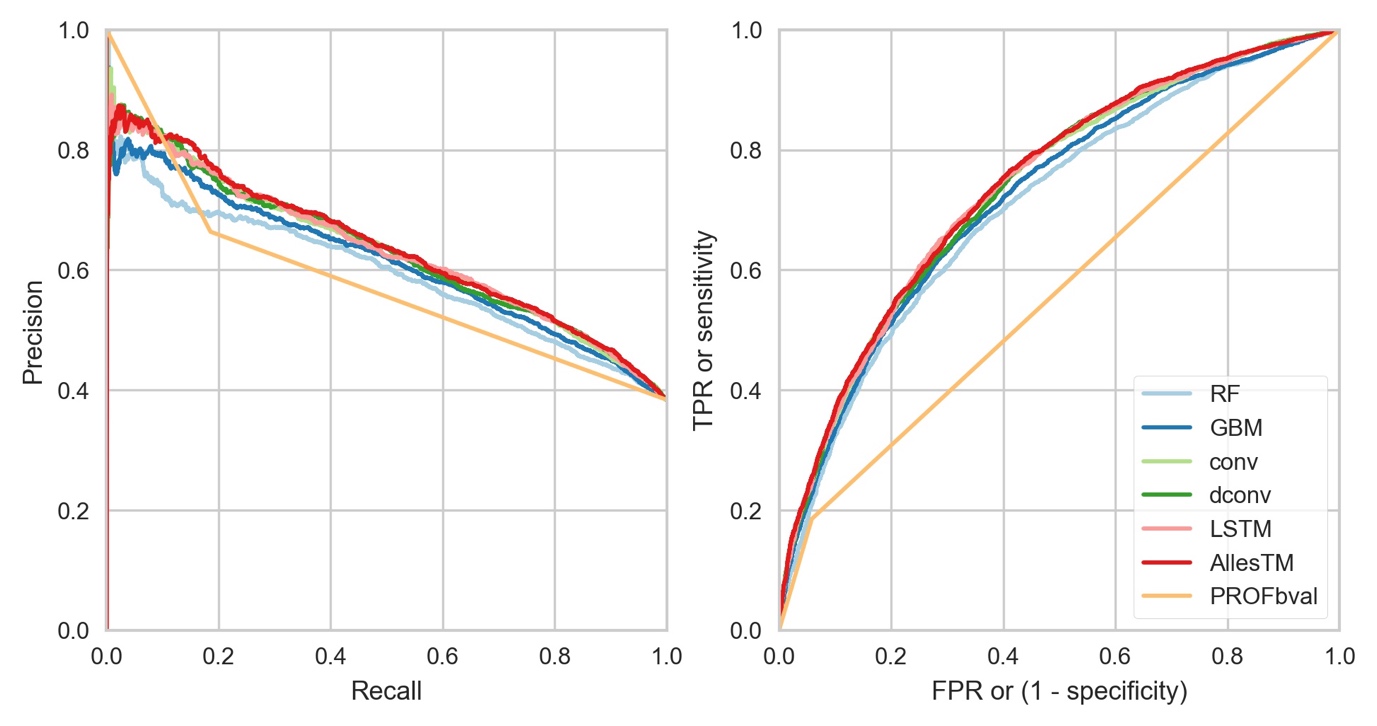


S8 Fig - Precision-recall and ROC curve of the predicted two-state flexibility on the independent test dataset.


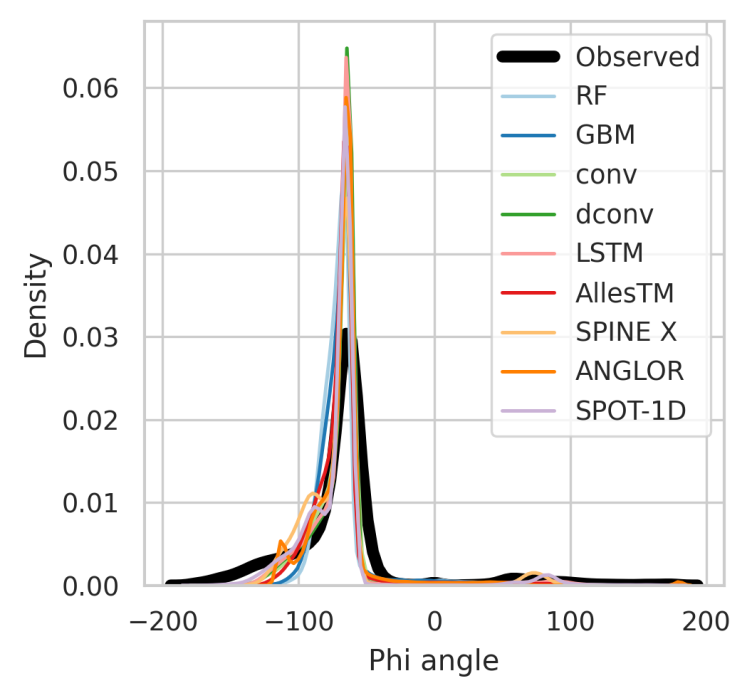


S9 Fig - Distribution of the observed and predicted ϕ angles on the cross-validation dataset.


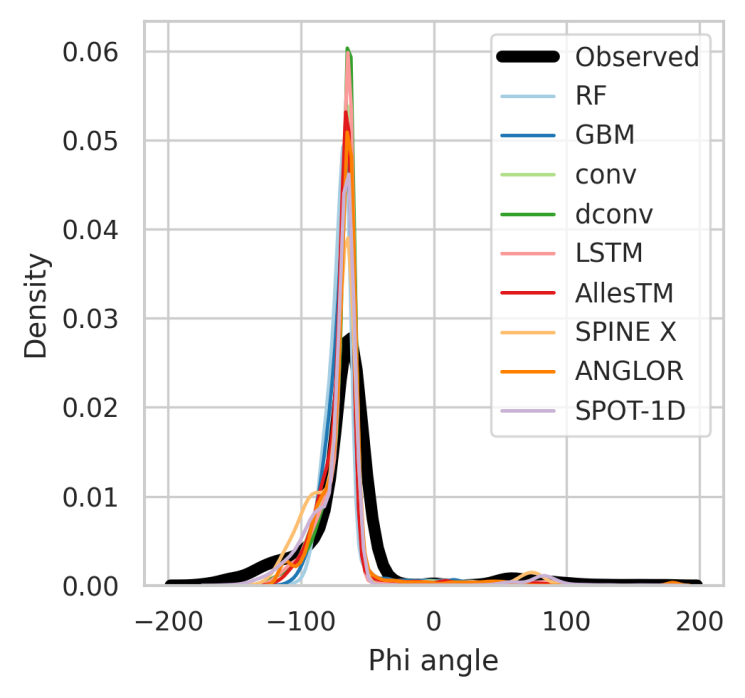


S10 Fig - Distribution of the observed and predicted ϕ angles on the independent test dataset.


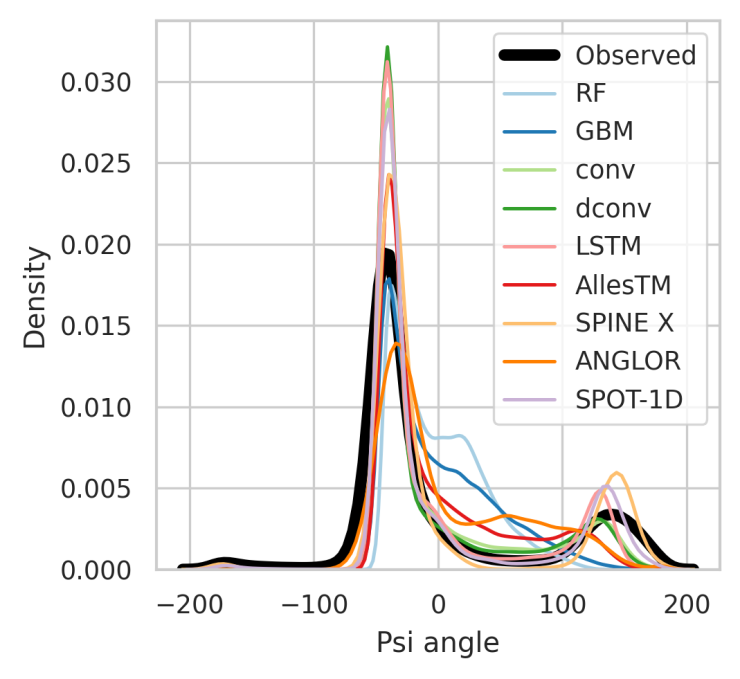


S11 Fig - Distribution of the observed and predicted ψ angles on the cross-validation dataset.


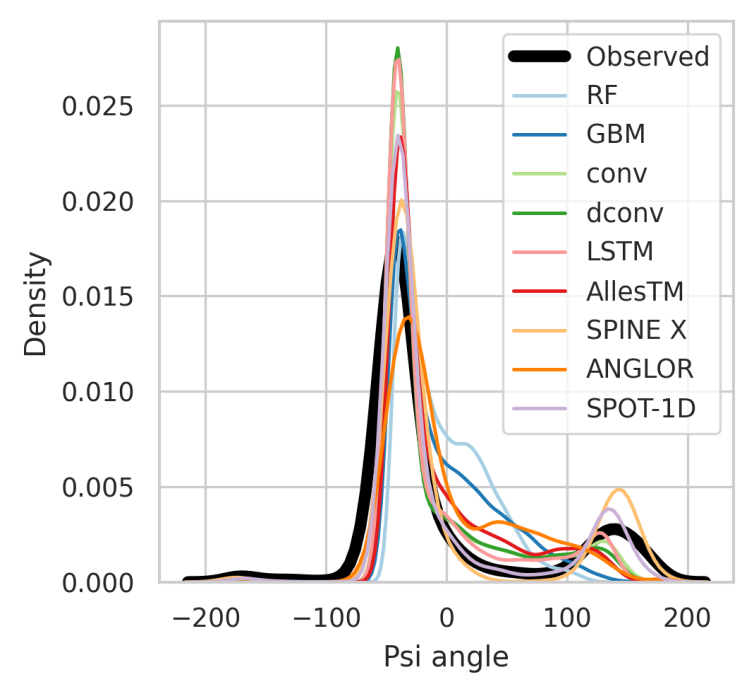


S12 Fig - Distribution of the observed and predicted ψ angles on the independent test dataset.


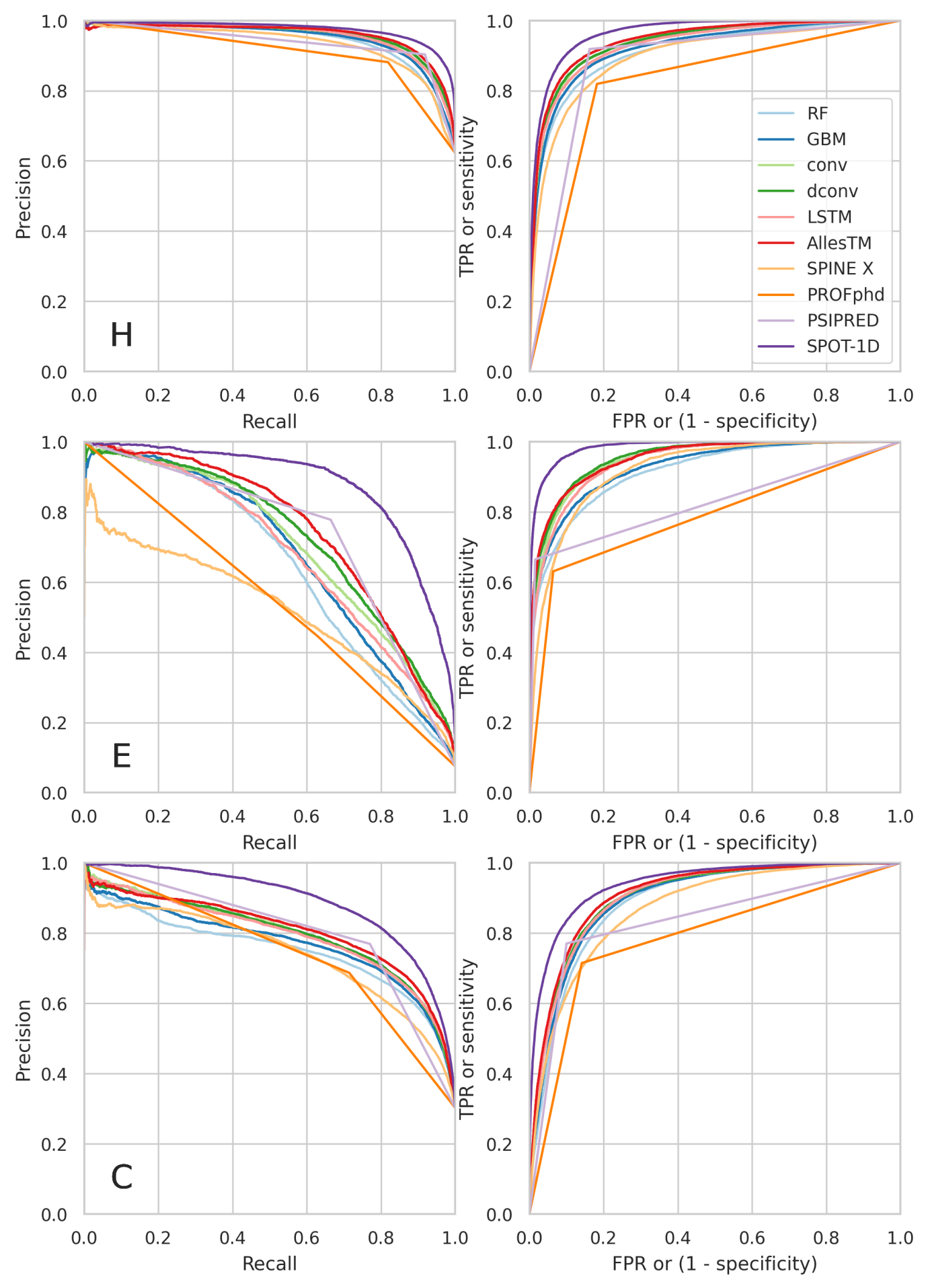


S13 Fig - Precision-recall and ROC curves of the predicted secondary structure on the cross-validation dataset.


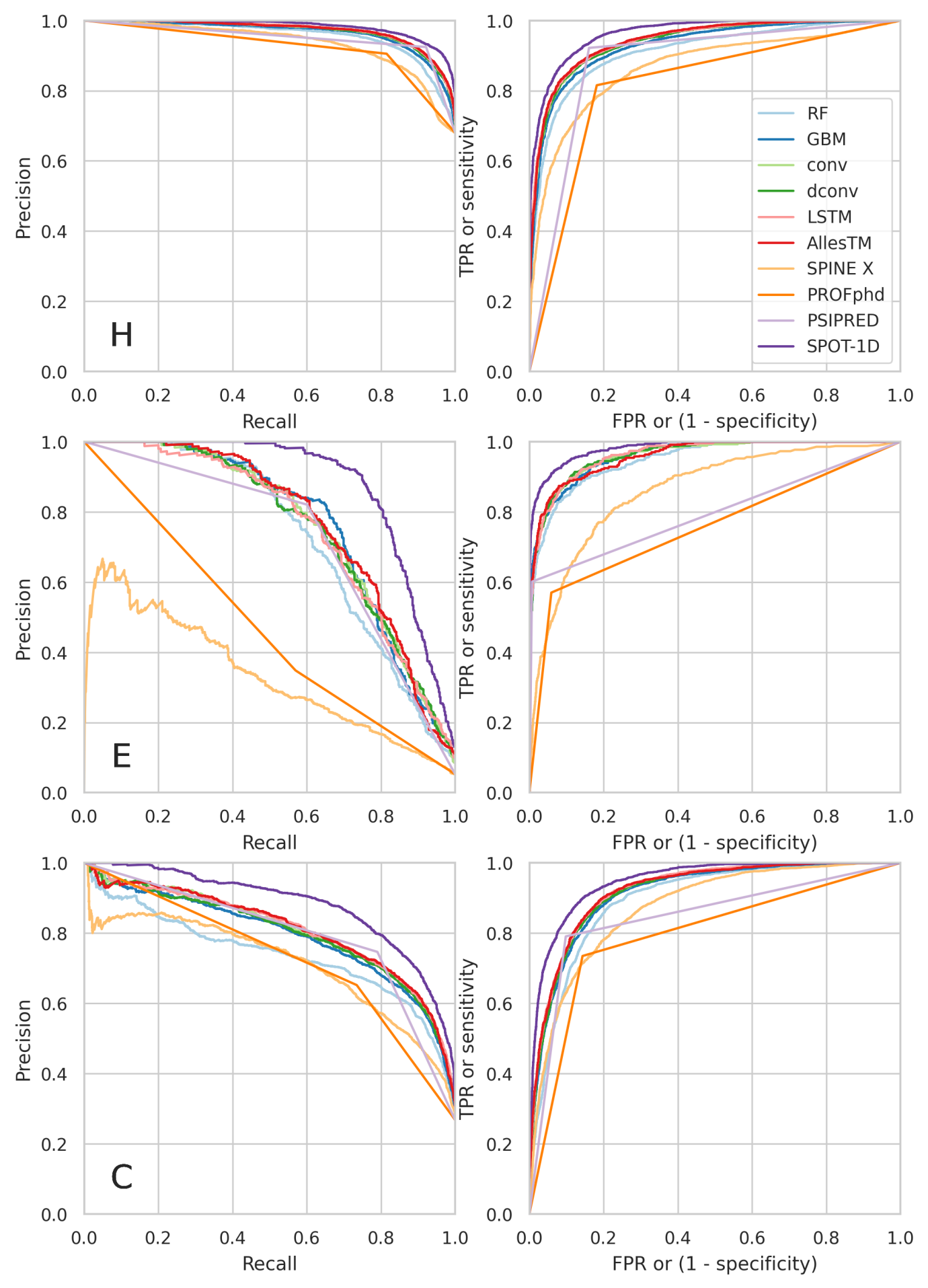


S14 Fig - Precision-recall and ROC curves of the predicted secondary structure on the independent test dataset.


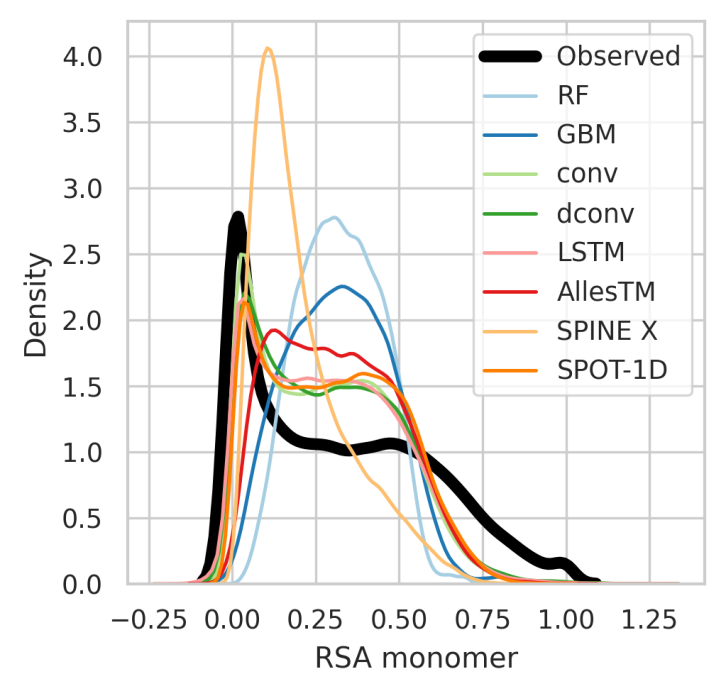


S15 Fig - Distribution of the observed and predicted relative solvent accessibility of monomers on the cross-validation dataset.


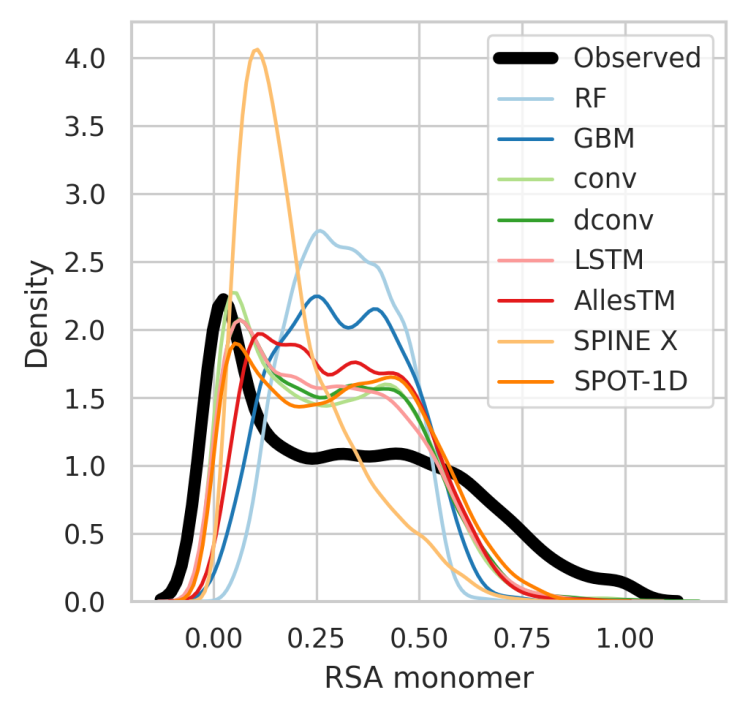


S16 Fig - Distribution of the observed and predicted relative solvent accessibility of monomers on the independent test dataset.


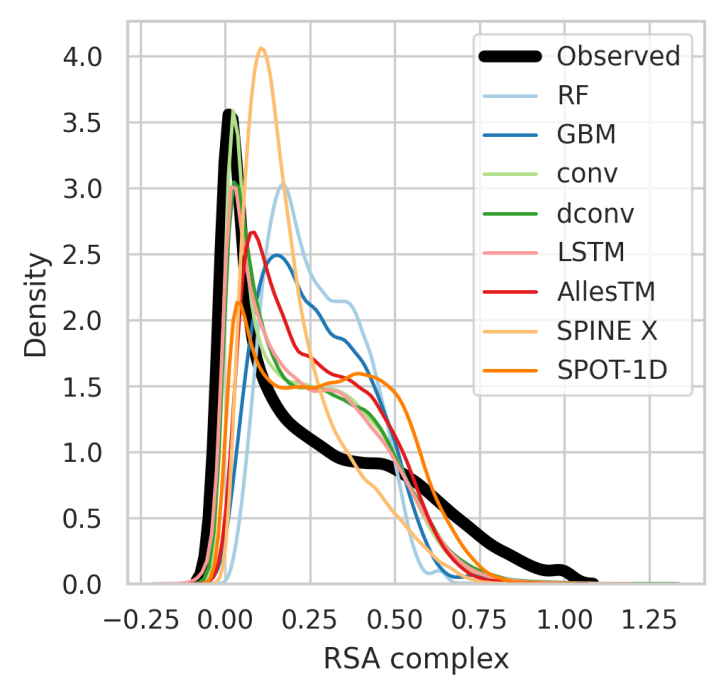


S17 Fig - Distribution of the observed and predicted relative solvent accessibility of protein chains in complexes on the cross-validation dataset.


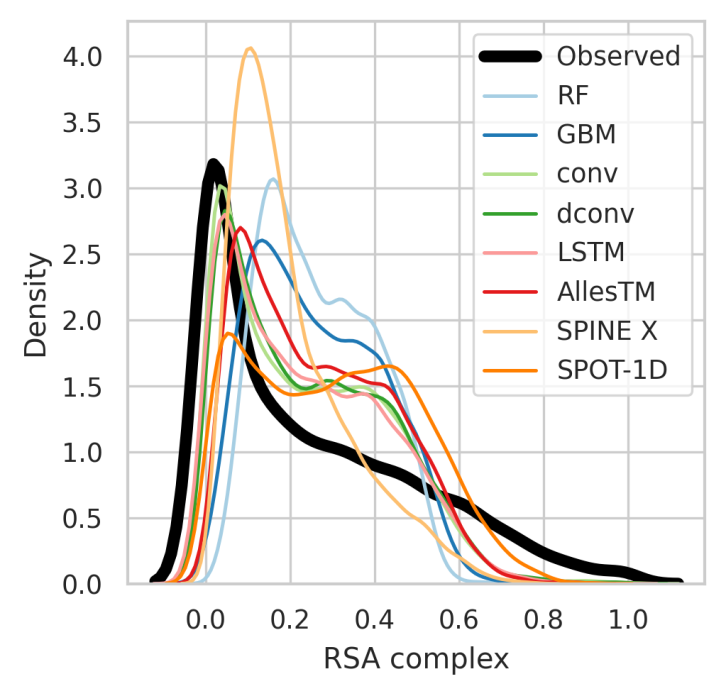


S18 Fig - Distribution of the observed and predicted relative solvent accessibility of protein chains in complexes on the independent test dataset.


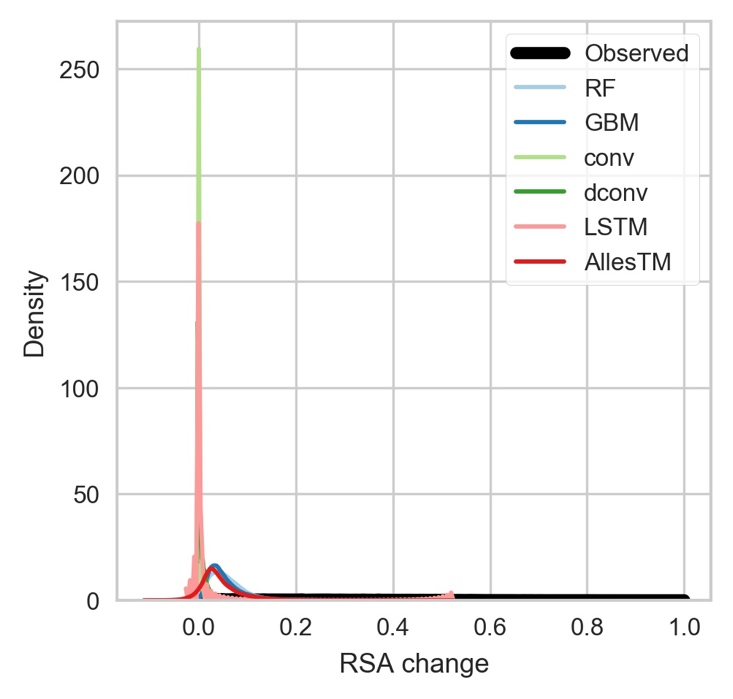


S19 Fig - Distribution of the observed and predicted difference in relative solvent accessibility between the bound and the unbound form of a protein chain on the cross-validation dataset.


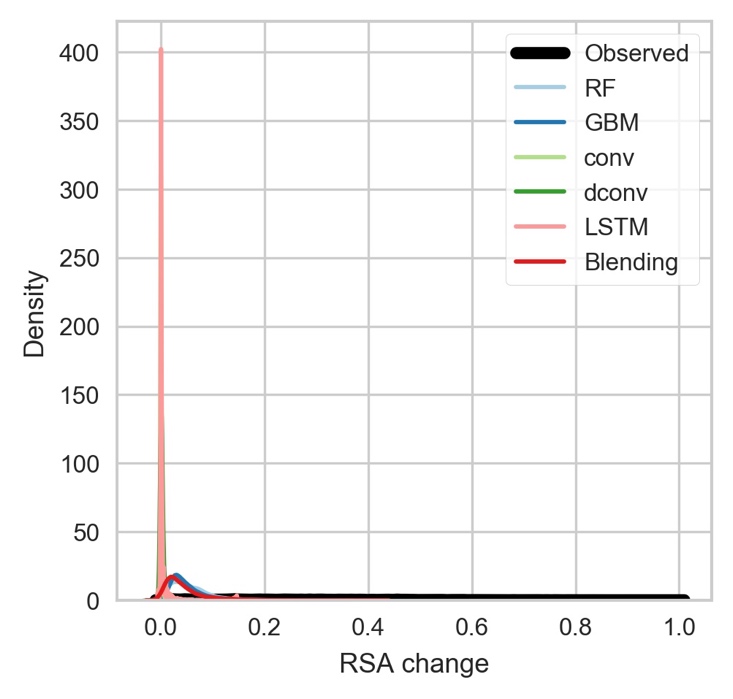


**S20 Fig - Distribution of the observed and predicted difference in relative solvent accessibility between the bound and the unbound form of a protein chain on the ind**
